# Enhanced removal of rare earth elements from aqueous media: exploring the potential of AM-3 and AM-4 titanosilicates

**DOI:** 10.1007/s11356-024-33063-w

**Published:** 2024-04-02

**Authors:** Joana C. Almeida, Cátia Sousa, Daniela S. Tavares, João Pinto, Bruno Henriques, Zhi Lin, João Rocha, Eduarda Pereira

**Affiliations:** 1 Chemistry Department and CICECO-Aveiro Institute of Materials, University of Aveiro, Campus de Santiago, 3810-193 Aveiro, Portugal; 2 Chemistry Department and LAQV-REQUIMTE, University of Aveiro, Campus de Santiago, 3810-193 Aveiro, Portugal; 3 Central Laboratory of Analysis, University of Aveiro, Campus de Santiago, 3810-193 Aveiro, Portugal

**Keywords:** Rare earth elements, Box-Behnken design, Process optimization, Sorption, Titanosilicates

## Abstract

This study investigates the impact of three key variables on the performance of nanoporous AM-3 and layered AM-4 titanosilicates in removing nine REEs (Y, La, Ce, Pr, Nd, Eu, Gd, Tb, and Dy) from natural mineral water and identifies optimal operational conditions using Response Surface Methodology (RSM). The experimental conditions were determined by a Box-Behnken Design of 3 factors-3 levels (pH 4, 6, and 8; sorbent dose 20, 100, and 180 mg/L; and element concentration 1, 3, and 5 μmol/L). Three-dimensional response surfaces were used to assess the linear, quadratic, and interaction influences of each factor on the REEs’ removal percentage. The pH was the most significant factor in the removal process using AM-3, while the sorbent dose was more important for AM-4. The results highlighted the sorbents’ strong capacity for REE removal. The optimal operating conditions obtained by RSM were applied to aqueous solutions with salinity 10 (common in coastal and transitional systems) and 30 (average seawater salinity). The results showed that AM-3 has a strong potential for removing REEs in solutions with salinity 10 and 30, while AM-4 was less efficient due to competition between REEs and other ions present in the solution.

**Supplementary Information:**

The online version contains supplementary material available at 10.1007/s11356-024-33063-w.

## Introduction

Rare earth elements (REEs) are considered critical resources for the European Union due to their vital role in various applications. The International Union of Pure and Applied Chemistry (IUPAC) classifies REEs as a group of 15 lanthanides (La, Ce, Pr, Nd, Pm, Sm, Eu, Gd, Tb, Dy, Ho, Er, Tm, Yb, and Lu), along with Sc and Y. These elements are crucial for numerous applications, particularly in the automotive industry, where they contribute to alternative power and energy-saving technologies. La and Ce are found in hybrid batteries and catalytic converters, and Nd, Dy, Pr, and Tb are components of hybrid electric motors and generators, while Eu, Tb, and Y are utilized in color LCD screens (Malhotra et al. 2020).

As a consequence of their increased use, the demand for REEs is anticipated to grow. Currently, mining serves as the primary production source for these elements. However, extraction processes, such as pyrometallurgical and hydrometallurgical methods, demand substantial energy and water resources and generate considerable amounts of chemical waste (Binnemans et al. 2013). This leads to adverse environmental and human health impacts. Moreover, rapid technological advancements have caused these elements to increasingly emerge as contaminants (World Economic Forum 2019). Hence, it is crucial to develop efficient techniques for REE removal and recovery from water sources to reduce mining reliance and preserve natural resources.

Numerous methods have been proposed for removing chemical elements from aqueous solutions, with sorption emerging as the most promising and widely used technique. The sorption process offers several benefits, including versatility, simplicity, cost-effectiveness, and high efficiency (Anastopoulos et al. 2016). Numerous studies have highlighted the high sorption capacity of various sorbent materials for different REEs. Examples of these materials include live algae (Jacinto et al. 2018), bacteria (Liang and Shen 2022), activated carbon and silica compounds (Ramasamy et al. 2018), sericin/alginate particles (Da Costa et al. 2021), and by-pass cement dust (Ali et al. 2011). Nevertheless, certain limitations have been observed. For instance, in the case of biosorbents like algae, prolonged sorption contact times are needed to achieve high removal efficiencies. Additionally, some studies are conducted in a simple matrix, which prevents the evaluation of the impact of ion competition on sorption. Recently, zeolite-type materials have demonstrated significant potential for REE removal (Thakkar et al. 2019) due to their high selectivity, removal efficiency, rapid sorption kinetics and ease of removal and recovery.

Titanosilicates are a class of inorganic materials which are part of the larger family of zeolites and zeolite-like materials, known for their unique framework structures with uniform pore sizes, high surface area, and remarkable ion-exchange properties (Sankar et al. 1996; Rocha and Anderson 2000). The negatively charged frameworks of microporous titanosilicates, built up of [TiO_6_], rarely of [TiO_5_], polyhedra and [SiO_4_] tetrahedra, are balanced by extra-framework cations, such as Na^+^ or K^+^. ETS-10 [(Na,K)_2_TiSi_5_O_13_·4H_2_O] is the most representative member of this family of materials (Anderson et al. 1994).

While the ion-exchange properties of titanosilicates have been much studied (Ferreira et al. 2009; Lopes et al. 2009), only a few publications have addressed the removal and recovery of REEs (Oleksiienko et al. 2017). Recently, ETS-10 was used in the selective recovery of Nd(III) from Ni − Nd acidic aqueous solutions and Nd − Dy acidic aqueous solutions, generated during the recycling of NiMH batteries and NdFeB permanent magnets, respectively (Thakkar et al. 2019).

This study focuses on evaluating two titanosilicates as sorbent materials for REE removal from aqueous solutions and optimizing the sorption process. AM-3 (Aveiro-Manchester number 3) is a synthetic nanoporous titanosilicate, analogous to the mineral penkvilksite-2*O*, with an ideal formula of Na_2_TiSi_4_O_11_·2H_2_O. Its structure comprises SiO_4_ tetrahedral chains interconnected by individual TiO_6_ octahedral units, creating a three-dimensional framework that features 6-ring channels which are partially occupied by Na^+^ cations and water molecules (Lin et al. 1997). On the other hand, AM-4 (Aveiro-Manchester number 4, Na_3_(Na,H)Ti_2_O_2_[Si_2_O_6_]_2_·2H_2_O) is composed of TiO_6_ (M) octahedra and SiO_4_ (T) tetrahedra, which form layers perpendicular to the [001] direction. Each layer consists of a five-tier sandwich structure of T-M-T-M-T. Na^+^ cations and water molecules are located between the layers, with Na^+^ cations also found within small cages inside the layers (Dadachov et al. 1997). Hence, the main difference between the two materials is that AM-3 exhibits a 3D framework while AM-4 has a 2D layered structure. Both titanosilicates have not been previously tested for REE removal.

Design of Experiments (DoE) and Response Surface Methodology (RSM) are powerful statistical modeling techniques that have been widely used to optimize sorption processes (Ferreira et al. 2007; Witek-Krowiak et al. 2014; Fabre et al. 2021). DoE is used to study the effects of experimental variables, also known as factors, on the sorption process and assesses their significant impact on the response variable in an experiment. Employed in the initial stages of experimental research, DoE helps to identify the most crucial factors to consider in subsequent studies. This approach enables researchers to optimize experimental conditions, minimize errors, and efficiently allocate resources while obtaining meaningful results (Witek-Krowiak et al. 2014). RSM is then applied to the experimental data generated by DoE to construct a model that describes the relationship between the factors and the target response. The quality of the RSM model is contingent upon the quality of the experimental data generated by DoE. The RSM model can be utilized to optimize the response variable by identifying the optimal levels of the factors that either maximize or minimize the response (Witek-Krowiak et al. 2014). The Box-Behnken design is a widely used method in RSM due to its ability to determine optimal conditions with high precision while requiring a reduced number of experiments. This design is particularly beneficial when investigating the effects of multiple factors on a response variable within a specified experimental region (Ferreira et al. 2007).

The primary objectives of this study are as follows: (i) to assess the influence of various experimental conditions (pH, sorbent dose, and initial REE concentration) on the removal of these elements from aqueous solutions using AM-3 and AM-4 titanosilicates, employing the Box-Behnken design; (ii) to develop a model that describes the process under the studied circumstances and determines the optimal conditions for REE removal by titanosilicates; and (iii) to compare the performance of the two titanosilicates in REEs sorption from high salinity matrices.

## Experimental section

### Material and reagents

All glass material used in the experiments was previously washed with nitric acid (HNO_3_ 25% v/v) obtained from Merck, Suprapur® 65%, for at least 24 h, and then rinsed with ultrapure water (Milli-Q water, 18 MΩ/cm). All chemicals used in this work were of analytical grade, obtained from chemical suppliers and used without additional purification. The standard solutions of Y (1001 ± 4 μg/mL in HNO_3_ 2%), La (1001 ± 5 μg/mL in HNO_3_ 2%), Ce (1016 ± 30 μg/mL in HNO_3_ 10%), Nd (1000 ± 3 μg/mL in HNO_3_ 2%), Eu (1005 ± 4 μg/mL in HNO_3_ 7%), and Gd (1000 ± 3 μg/mL in HNO_3_ 5%) were obtained from Inorganic Ventures. The standard solutions of Pr (1000 ± 4 μg/mL in HNO_3_ 2%), Dy (1000 ± 4 μg/mL in HNO_3_ 2%), and Tb (1000 ± 4 μg/mL in HNO_3_ 2%) were obtained from CPAchem.

### Synthesis of sorbent materials

#### AM-3 synthesis

An alkaline solution was prepared by mixing 40.45 g of sodium silicate solution (7.5–8.5 m/m% Na_2_O, 25.5–28.5 m/m% SiO_2_), 8.66 g of NaOH, 3.38 g of NaCl, 3.54 g of KCl, and 19.53 g of H_2_O. TiCl_3_ (37.11 g; 15% m/m solution of TiCl_3_ in 10% m/m HCl, Merck) was added to this solution and stirred thoroughly. Finally, 0.44 g of AM-3 seeds was added to the mixture. The resulting gel, with a composition of 5.2Na_2_O:0.7K_2_O:5.0SiO_2_:1.0TiO_2_:113H_2_O, was heated at 230 °C in Teflon in-lined autoclaved under autogeneous pressure for 7 days.

#### Typical AM-4 synthesis

1.88 g of TiO_2_ was dispersed well in 41.33 g of H_2_O with 3.40 g of NaOH. Then 16.32 g of sodium silicate solution was added and stirred thoroughly. Finally, 0.28 g of AM-4 seeds were added to the mixture. The resulting mixture, with a composition of 2.7Na_2_O:3.1SiO_2_:1TiO_2_:123H_2_O, was treated at 230 °C for 10 h or 200 °C for 1 day under autogeneous pressure.

### Structural and chemical characterization techniques

The powder X-Ray diffraction (PXRD) patterns of both samples were recorded on an Empyrean PANalytical diffractometer equipped with a Cu-Kα monochromatic radiation source. The crystal morphology of AM-3 and AM-4 was analyzed using scanning electron microscopy (SEM) on a Hitachi SU-70 SEM microscope with a Bruker Quantax 400 detector operating at 20 kV.

### Experimental design

The sorption experiments were carried out to evaluate the sorption performance of two different sorbent materials (titanosilicates AM-3 and AM-4) on a solution of REEs for a contact period of 48 h. The working solutions were prepared by diluting a specific volume of commercial stock solutions of 9 REEs (Y, La, Ce, Pr, Nd, Eu, Gd, Tb, and Dy) in natural mineral water (composition given in Supporting Information). The use of natural mineral water intends to simulate the tap water that industries use in their processes. Different amounts of titanosilicates (20, 100, and 180 mg/L) were placed in contact with three different REE concentration solutions (1, 3, and 5 μmol/L), representing levels found in some aquatic systems (Åström 2001). The pH was adjusted to 4, 6 or 8 with HNO_3_ 2% (v/v) and NaOH (1 and 10 mol/L). Solution samples were taken after 1 and 6 h following titanosilicate addition, immediately centrifuged at 5000 rpm for 3 min and then acidified with HNO_3_ (65%) to ensure pH < 2 and stored at 4 °C for REEs quantification. Control solutions (natural mineral water containing only the chemical elements, in absence of sorbent) were always run in parallel with the assays to evaluate potential experimental REEs losses (by precipitation or sorption on the vessels walls) or contamination.

The removal efficiency of REEs by AM-3 and AM-4 in high salinity solutions was further investigated using real seawater (composition given in Supporting Information) as the basis for REE solutions. Seawater with varying salinities was prepared by diluting filtered seawater with ultrapure water to achieve the desired salinity levels. Since industrial effluents typically have an ionic strength greater than zero, the use real seawater intends to mimic a matrix with a higher ionic strength.

### REE elemental analysis

The quantification of REEs in solution was performed by Inductively Coupled Plasma Mass Spectrometry (ICP-MS), with a Thermo ICP-MS X Series equipped with a Burgener nebulizer. Calibration curves used five standards with concentrations between 0.1 and 100 μg/L prepared by the dilution of the commercial certified stock solutions into acidified water (HNO_3_ 1% v/v). Only calibration curves with a correlation coefficient above 0.999 were accepted. The limit of quantification was considered the lowest standard of the calibration curve (0.1 μg/L). The coefficient of variation between sample replicates (at least, three replicates were investigated) did not exceed 5%.

The removal efficiency, *R* (%), for each REE studied in each sorbent material, AM-3 and AM-4, was calculated as follows (Eq. (1)):

1 $$\mathrm{Removal\;}(\mathrm{\%})=\frac{{(C}_{0}-{C}_{t})}{{C}_{0}}*100$$

Where ${C}_{0}$ (μg/L) is the initial concentration of REEs in solution and ${C}_{t}$ (μg/L) is the concentration of REEs at time $t$.

### Response Surface Methodology

RSM is a robust statistical technique employed to model and refine the relationship between independent variables, or factors, and the target response under investigation. By utilizing experimental data obtained from DoE, RSM facilitates the development of a mathematical model that accurately forecasts the response as a function of the examined factors (Witek-Krowiak et al. 2014).

In this study, a Box-Behnken design with three factors and three levels was employed. This method enables the identification of the most pertinent experiments for evaluating various factors, while substantially reducing the number of experiments required, thereby enhancing the efficiency of the experimental procedure (Fabre et al. 2021). The three independent variables investigated were solution pH, sorbent dosage, and initial concentration of REEs, with each variable being assigned three equidistant values (− 1; 0; 1), as displayed in Table 1. The effects of these variables were assessed on the performance of two sorbent materials, AM-3 and AM-4, with the response of interest being the percentage removal of titanosilicates. To increase the accuracy, three replicates were conducted at the central point. The experiments generated by the Box-Behnken design are presented in Table 2, with identical conditions for both sorbents tested.

**Table 1:** Experimental conditions used for the three different factors studied and the three level conditions

Variable	Level		
	− 1	0	1
pH	4	6	8
Dose of sorbent (mg/L)	20	100	180
Initial concentration of REEs (µmol/L)	1	3	5

**Table 2:** Description of the experimental conditions according to the Box-Behnken design

Experiment	pH	Dose of sorbent (mg/L)	Initial concentration of REEs (µmol/L)
1	8	20	3
2	6	100	3
3	4	100	5
4	6	20	1
5	8	100	5
6	8	100	1
7	6	100	3
8	6	100	3
9	6	180	5
10	6	180	1
11	4	20	3
12	6	20	5
13	4	100	1
14	4	180	3
15	8	180	3

RSM uses codified values for variables instead of the real values, to simplify the analysis. The input variables are transformed to have a mean of zero and a standard deviation of one. The codified values of the independent variables (${X}_{k}$) are calculated by the equation represented below:

2 $${X}_{k}=\frac{{(x}_{k}-{x}_{0})}{{\Delta x}_{k}}$$

where ${x}_{k}$ corresponds to the uncodified value of the independent variables, ${x}_{0}$ is the variable value at its center point, and ${\Delta x}_{k}$ is the step change between levels for the $k$ variable.

The effects of variables (linear, quadratic, and combined effects) are described by RSM through a second order polynomial function, expressed by the Eq. (3), that allows to predict the optimized conditions:

3 $$Y={\mathrm\beta}_0+\sum\limits_{i=1}^k{\mathrm\beta}_iX_i+\sum\limits_{j=1}^k{\mathrm\beta}_{ii}X_i^2+\sum\limits_{i=j}^k{\mathrm\beta}_{ij}X_iX_j$$

where $Y$ is the response variable studied, ${X}_{i}$, ${X}_{j}$, … ${X}_{k}$ are codified values of the independent variables, ${\beta }_{0}$ is a constant, and ${\beta }_{i}$, ${\beta }_{ii}$, and ${\beta }_{ij}$ are the regression coefficients for the linear, quadratic, and interaction terms (Ferreira et al. 2007; Witek-Krowiak et al. 2014; Fabre et al. 2021).

The results were obtained using the software Design-Expert version 13 (Stat-Ease Inc.) and Minitab Statistical Software version 20. The significance of the factors was assessed by Analysis of variance (ANOVA), and the interactions between them were evaluated using Fisher’s test and its associated probability $p(F)$. To check the goodness of the adjustments, the coefficient of determination, ${R}^{2}$, and the adjusted coefficient of determination, ${R}_{adj}^{2}$ (Eqs. (4) and (5), respectively) were used:

4 $${R}^{2}=1-\frac{{\sum (\widehat{{y}_{i}}-{y}_{i})}^{2}}{{\sum ({y}_{i}-\overline{y })}^{2}}$$

5 $${R}_{adj}^{2}=1-\left(1-{R}^{2}\right)\frac{{(N}_{DP}-1)}{{(N}_{DP}-{N}_{P}-1)}$$

where $\widehat{{y}_{i}}$ are the values calculated by the model obtained, ${y}_{i}$ are the experimental values, $\overline{y }$ is the mean of the experimental values, ${N}_{DP}$ is the number of experimental data points, and ${N}_{P}$ is the number of parameters.

## Results and discussion

### Characterization of titanosilicates AM-3 and AM-4

**Fig. 1:**
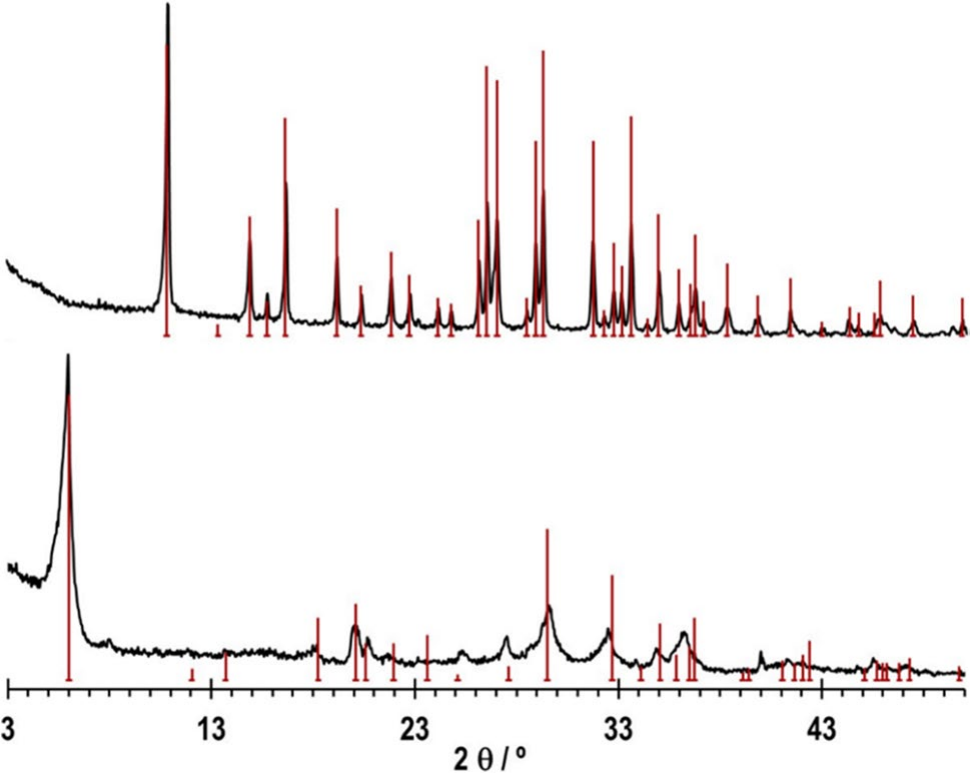
XRD diffractograms of titanosilicates AM-3 and AM-4. The bars depict reflections calculated from the crystal structure

The powder XRD patterns of pristine AM-3 and AM-4, shown in Fig. 1, are in accord with the published ones (Lin et al. 1997). The bars in Fig. 1 depict the powder XRD reflections calculated from the crystalline structure of AM-3 and AM-4. No extra peak is observed, indicating the high purity of both materials.

**Fig. 2:**
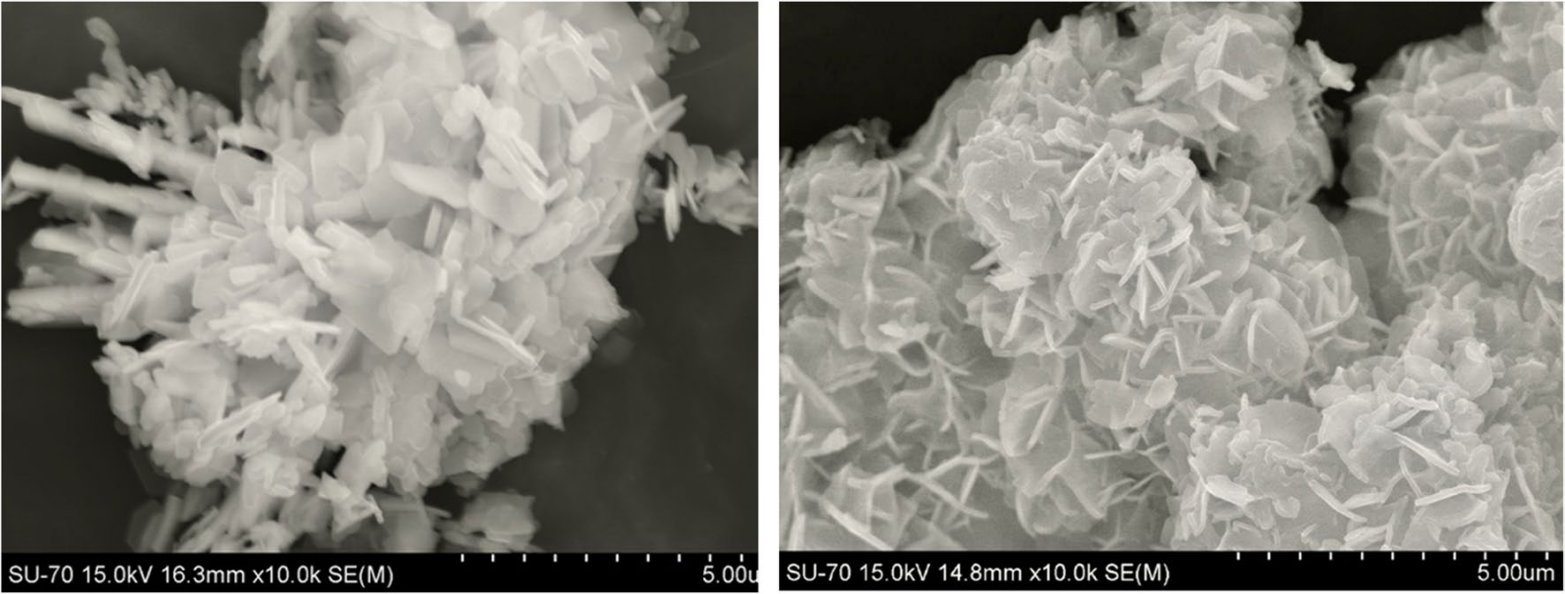
SEM images of titanosilicates AM-3 (left) and AM-4 (right)

SEM (Fig. 2) reveals that both materials contain plate like crystals, with sizes ranging from ca. 0.5 to 1.4 µm (AM-3) and 1.7 to 1.3 µm (AM-4) and widths of 0.05–0.22 µm (AM-3) and 0.07–0.11 µm (AM-4). We have previously shown that both materials do not adsorb significant amounts of nitrogen, but they do adsorb water in relatively large amounts: the water adsorption isotherms of AM-3 and AM-4 are of type I with maximum water uptakes of 0.117 and 0.070 g/g_solid_, respectively (Lin et al. 1997).

### Development of regression model equations

The DoE described in Table 2 was performed for 1 and 6 h, and the removal (%) of the different REEs is presented in Tables 3 and 4.

**Table 3:** Removal (%) of Y, La, Ce, Pr, Nd, Eu, Gd, Tb, and Dy, at 1 and 6 h, using the sorbent AM-3, under the experimental conditions presented in Table 2

Experiment	Removal (%)																	
	Y		La		Ce		Pr		Nd		Eu		Gd		Tb		Dy	
	1h	6h	1h	6h	1h	6h	1h	6h	1h	6h	1h	6h	1h	6h	1h	6h	1h	6h
1	67	69	80	82	80	82	78	81	79	81	78	80	77	80	77	80	76	78
2	3	5	2	4	3	7	3	7	4	8	8	12	7	8	8	11	7	11
3	1	2	0	1	1	0	1	0	1	1	3	3	2	3	3	2	2	2
4	0	1	0	2	0	4	0	5	0	5	0	7	2	6	3	8	2	8
5	81	83	86	92	86	93	86	92	85	92	86	92	84	91	88	93	85	90
6	55	55	63	64	68	68	65	66	66	66	65	65	66	66	65	66	65	65
7	0	3	0	4	2	8	2	9	2	10	5	13	3	10	6	13	6	12
8	2	4	2	5	4	9	4	9	4	10	6	12	4	9	7	11	9	12
9	8	8	6	7	7	9	8	9	8	9	13	11	11	10	14	12	14	12
10	12	12	9	12	15	21	16	22	17	22	29	33	25	29	32	36	32	35
11	0	3	0	0	1	2	0	2	0	2	1	4	1	3	1	2	1	2
12	0	0	0	0	0	1	0	2	0	2	0	4	0	2	0	2	0	0
13	0	0	1	0	1	1	1	0	2	1	3	3	3	2	4	5	5	6
14	2	3	2	1	2	1	2	1	2	2	1	4	1	3	2	5	2	5
15	76	72	84	82	84	82	83	80	83	81	83	80	82	80	83	80	82	79

**Table 4:** Removal (%) of Y, La, Ce, Pr, Nd, Eu, Gd, Tb, and Dy, at 1 and 6 h, using the sorbent AM-4, under the experimental conditions presented in Table 2

Experiment	Removal (%)																	
	Y		La		Ce		Pr		Nd		Eu		Gd		Tb		Dy	
	1h	6h	1h	6h	1h	6h	1h	6h	1h	6h	1h	6h	1h	6h	1h	6h	1h	6h
1	44	54	47	56	47	57	48	57	47	57	47	57	46	57	47	58	46	57
2	63	69	70	77	74	81	74	80	72	79	75	80	72	78	74	80	73	79
3	10	56	12	53	11	64	10	66	9	65	9	70	7	65	8	69	8	69
4	21	48	24	57	26	62	26	61	23	59	28	62	26	59	27	61	25	59
5	82	85	81	83	81	83	81	83	81	83	81	83	81	83	85	87	82	84
6	42	58	53	58	44	56	53	58	54	58	54	59	55	60	54	60	57	63
7	53	71	60	79	64	84	64	83	63	82	65	84	62	81	64	83	63	82
8	59	70	65	77	71	83	71	82	70	81	73	83	70	81	72	82	71	81
9	41	91	85	92	67	90	85	92	83	91	78	89	78	89	75	89	82	90
10	63	86	82	85	86	84	81	84	81	85	66	84	61	85	43	85	82	85
11	26	26	2	2	0	0	1	1	1	1	0	0	0	0	0	0	2	2
12	3	10	4	13	7	24	9	27	7	25	10	31	6	23	9	26	9	24
13	72	71	73	72	72	70	72	71	71	70	72	71	71	71	71	71	72	71
14	81	85	75	81	74	79	75	80	73	79	73	79	71	77	72	78	73	78
15	19	41	42	65	39	43	43	64	43	65	44	63	45	63	46	61	49	68

For AM-3, the highest values of the removal percentage were achieved in the Experiment 5 (initial REE concentration of 5 µmol/L, pH 8 and titanosilicate dosage of 100 mg/L), with 81–88% REE removal after 1 h and 83–93% REE removal after 6 h. The maximum removal achieved by titanosilicate AM-4 for most of the elements was observed in Experiment 9 (initial REE concentration of 5 µmol/L, pH 6 and titanosilicate dosage of 180 mg/L), with removal percentages of 41% for Y, 67% for Ce and 75–83% for the other REEs after 1 h, and 90–92% REE removal after 6 h. These results indicate that AM-4 supports higher concentrations of REEs and lower pH in water than AM-3, which in turn performs better at higher pH. Additionally, for AM-3, extending the experimental duration did not yield any benefits in terms of percentage removal. This finding is particularly significant for industrial applications, as longer contact times result in increased energy consumption and labor costs, making shorter sorption processes more appealing.

The removal rates observed in various experiments exhibited considerable variability based on the tested parameters (pH, sorbent dosage, and initial REE concentration), necessitating the use of Response Surface Methodology for experimental parameter optimization. Consequently, a quadratic model was fitted to the experimental values after obtaining the response data for each trial.

**Fig. 3:**
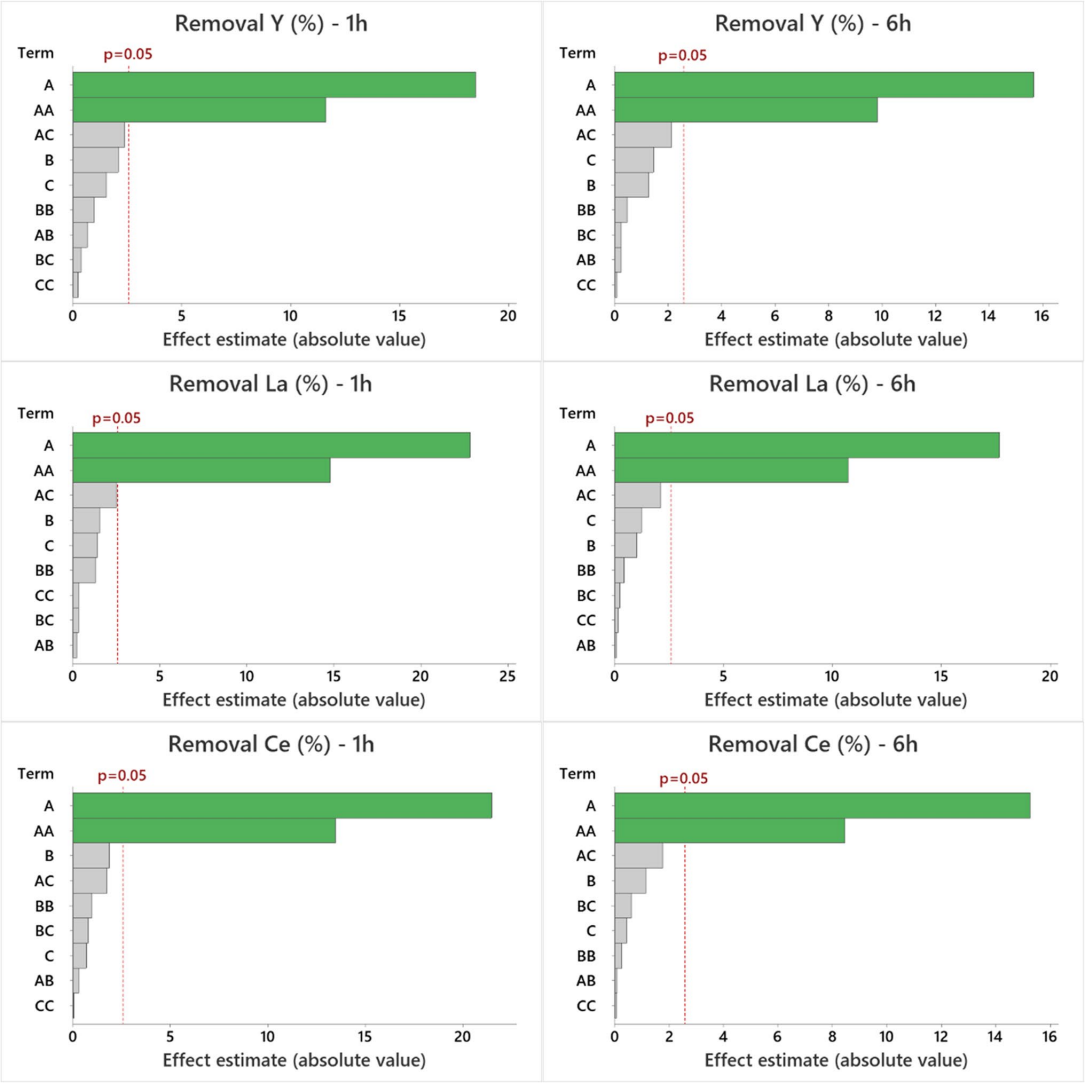
Pareto chart displaying the effects of variables on the studied response (removal percentage of Y, La, and Ce) at 1 and 6 h for AM-3. In the figure, **A** represents the solution pH, **B** denotes the sorbent dosage (mg/L), and **C** signifies the initial concentration of REEs (µmol/L). Variables with values below the dashed line are not significant

**Fig. 4:**
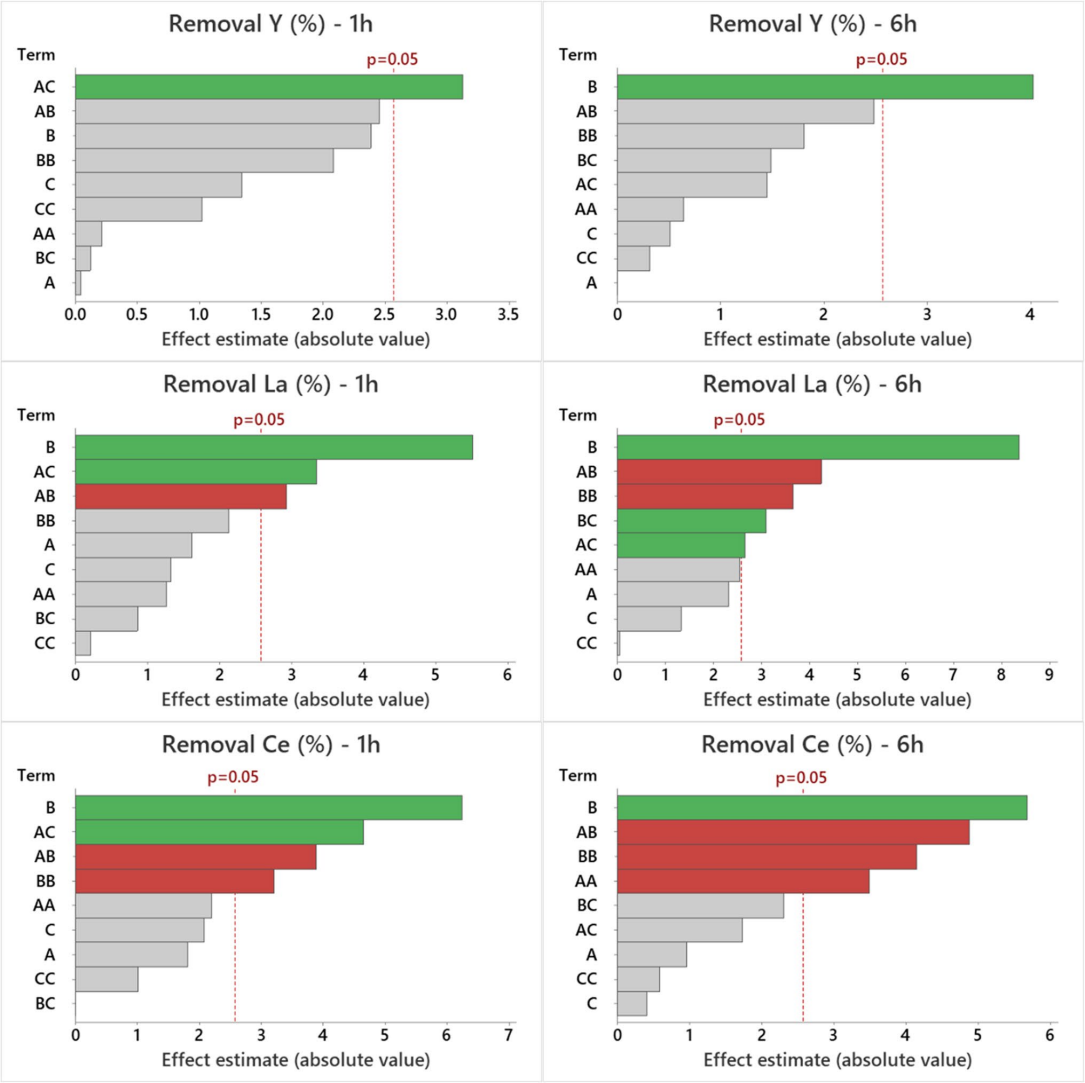
Pareto chart displaying the effects of variables on the studied response (remova l% of Y, La, and Ce) at 1 and 6 h for AM-4. In the figure: **A** represents the solution pH, **B** denotes the sorbent dosage (mg/L), and **C** signifies the initial concentration of REEs (µmol/L). Variables with values below the dashed line are not significant

The effects of variables (linear, quadratic, and combined effects) on the REE removal percentage, calculated with a 95% confidence level, are displayed in Figs. 3 and S1 for AM-3 and Figs. 4 and S2 for AM-4. In the Pareto chart, the variables pH, sorbent dosage, and initial REE concentration are denoted by the letters A, B, and C, respectively. Factors surpassing the red line significantly affect the studied response (*p*-value < 0.05); variables depicted with green bars positively impact the response (it increases as the variable value rises), while red bars signify a negative impact (the response decreases as the variable value increases), and grey bars represent non-significant variables. The p-values obtained for the fitted models are provided in the Supplementary data (Tables S1 and S2).

Regarding the removal percentage achieved by titanosilicate AM-3 (Figs. 3 and S1), it is evident that the impact of variables remains unchanged with time and element, with only the “pH” factor being significant. The linear and quadratic effects of pH contribute positively, indicating that a higher solution pH leads to an increased REE removal percentage.

Examining the Pareto charts for experiments using titanosilicate AM-4 (Figs. 4 and S2), the pattern of significant factors is markedly different compared to AM-3. Additionally, the variables affecting Y removal differ from those influencing the removal of other REEs. For Y, only one factor impacted removal: the interaction between “pH” and “initial concentration” was significant at 1 h, while the “sorbent dosage” factor was significant at 6 h, both displaying positive effects. For the other eight REEs studied, “sorbent dosage” is the most influential variable at both contact times, exhibiting a positive effect, followed by the interaction between “pH” and “sorbent dosage,” which has a negative effect. Other variables are either non-significant at 1 h and become significant at 6 h, or vice versa. For instance, the quadratic term of “pH” negatively affects the removal of Ce, Pr, Nd, and Eu only after 6 h. The quadratic term of “sorbent dosage” impacts the removal of Ce, Eu, Gd, and Tb after 1 h, and after 6 h, it also becomes significant for La, Pr, Nd, and Dy removal. Conversely, the interaction between “pH” and “initial concentration” affects the eight REEs after 1 h, while after 6 h, it is only significant for the removal of La, Gd, Tb, and Dy. Finally, the removal of La, Gd, Tb, and Dy is positively influenced by the interaction between “sorbent dosage” and “initial concentration” after 6 h.

In this study, the pH is an important factor in the removal efficiency by AM-3 due to competition between H^+^ and REE^3+^ ions for the exchange of the extra-framework Na^+^ cations. At low pH values, protonation of active sites on the sorbent occurs, inhibiting its ability to bind with REEs (Ali et al. 2011; Da Costa et al. 2021). For AM-4, the most important factor is the dose of sorbent. This is mainly due to the increase of mass leading to a higher number of binding sites available for sorption of REEs, improving the sorption efficiency (Ali et al. 2011).

Based on the insights provided by the Pareto charts, only significant variables were incorporated to generate the reduced models. Tables 5 and 6 display the reduced models for AM-3 and AM-4 in terms of the real values of the independent variables. The coefficient of determination (*R*^2^) and the adjusted coefficient of determination (*R*^2^_adj_) indicate the quality of the fits between the experimental and calculated data. AM-3 exhibited high *R*^2^ values (0.9324–0.9793) that were very close to the *R*^2^_adj_ values (0.9212–0.9758), demonstrating a strong fit and robustness of the models. Consequently, these models can accurately predict the response. In comparison, the *R*^2^ values for AM-4 functions of Y (0.3452–0.4539) and the other eight REEs (0.7820–0.9149) were lower than those for AM-3, indicating a less accurate estimation of the response. Furthermore, the discrepancies between the *R*^2^ and *R*^2^_adj_ values obtained for Y (0.1666–0.4119) and for the other eight REEs (0.6949–0.8297) undermine the robustness of the models.

**Table 5:** Reduced models of the response studied and the respective *R*^2^ and *R*^2^ adjusted as function of the significant variables (*p*-value < 0.05) for AM-3

		1h	6h
Y	Response reduced model	Removal (%) = 185.18 − 77.79 pH + 7.92 pH*pH	Removal (%) = 183.54 − 76.54 pH + 7.79 pH*pH
	*R*^2^	0.9619	0.9618
	*R*^2^_adj_	0.9556	0.9555
La	Response reduced model	Removal (%) = 217.54 − 90.98 pH + 9.20 pH*pH	Removal (%) = 204.14 − 86.30 pH + 8.85 pH*pH
	*R*^2^	0.9768	0.9721
	*R*^2^_adj_	0.9730	0.9674
Ce	Response reduced model	Removal (%) = 210.57 − 88.28 pH + 8.99 pH*pH	Removal (%) = 182.32 − 78.03 pH + 8.17 pH*pH
	*R*^2^	0.9793	0.9683
	*R*^2^_adj_	0.9758	0.9630
Pr	Response reduced model	Removal (%) = 202.29 − 85.11 pH + 8.70 pH*pH	Removal (%) = 171.75 − 74.00 pH + 7.81 pH*pH
	*R*^2^	0.9731	0.9652
	*R*^2^_adj_	0.9686	0.9594
Nd	Response reduced model	Removal (%) = 202.25 − 85.00 pH + 8.69 pH*pH	Removal (%) = 173.57 − 74.34 pH + 7.83 pH*pH
	*R*^2^	0.9741	0.9651
	*R*^2^_adj_	0.9698	0.9592
Eu	Response reduced model	Removal (%) = 176.29 − 74.86 pH + 7.82 pH*pH	Removal (%) = 153.61 − 65.76 pH + 7.06 pH*pH
	*R*^2^	0.9462	0.9420
	*R*^2^_adj_	0.9372	0.9323
Gd	Response reduced model	Removal (%) = 182.82 − 77.34 pH + 8.02 pH*pH	Removal (%) = 169.68 − 72.16 pH + 7.61 pH*pH
	*R*^2^	0.9600	0.9523
	*R*^2^_adj_	0.9533	0.9444
Tb	Response reduced model	Removal (%) = 169.75 − 72.19 pH + 7.59 pH*pH	Removal (%) = 153.96 − 65.96 pH + 7.08 pH*pH
	*R*^2^	0.9385	0.9326
	*R*^2^_adj_	0.9283	0.9214
Dy	Response reduced model	Removal (%) = 166.00 − 70.63 pH + 7.44 pH*pH	Removal (%) = 153.64 − 65.49 pH + 7.00 pH*pH
	*R*^2^	0.9392	0.9324
	*R*^2^_adj_	0.9290	0.9212

**Table 6:** Reduced models of the response studied and the respective R^2^ and R^2^ adjusted as function of the significant variables (p-value < 0.05) for AM-4

		1h	6h
Y	Response reduced model	Removal (%) = 172.39 − 19.25 pH − 42.13 concentration + 6.38 pH*concentration	Removal (%) = 35.62 + 0.26 mass
	*R*^2^	0.3452	0.4539
	*R*^2^_adj_	0.1666	0.4119
La	Response reduced model	Removal (%) = 32.82 − 0.69 pH + 1.05 mass − 36.50 concentration − 0.12 pH*mass + 5.56 pH*concentration	Removal (%) = 10.86 + 6.06 pH + 1.19 mass − 26.41 concentration − 0.0023 mass*mass − 0.11 pH*mass + 2.75 pH*concentration + 0.080 mass*concentration
	*R*^2^	0.8257	0.9149
	*R*^2^_adj_	0.7288	0.8297
Ce	Response reduced model	Removal (%) = 29.76 − 2.19 pH + 1.57 mass − 40.62 concentration − 0.0026 mass*mass − 0.13 pH*mass + 6.12 pH*concentration	Removal (%) = − 226.91 + 68.83 pH + 1.76 mass − 4.39 pH*pH − 0.0033 mass*mass − 0.15 pH*mass
	*R*^2^	0.8972	0.8604
	*R*^2^_adj_	0.8201	0.7828
Pr	Response reduced model	Removal (%) = 32.49 − 0.34 pH + 1.05 mass − 36.69 concentration − 0.12 pH*mass + 5.62 pH*concentration	Removal (%) = − 181.43 + 55.88 pH + 1.49 mass − 3.49 pH*pH − 0.0027 mass*mass − 0.11 pH*mass
	*R*^2^	0.8077	0.8639
	*R*^2^_adj_	0.7009	0.7882
Nd	Response reduced model	Removal (%) = 31.74 − 0.38 pH + 1.03 mass − 36.44 concentration − 0.12 pH*mass + 5.56 pH*concentration	Removal (%) = − 175.49 + 53.51 pH + 1.47 mass − 3.30 pH*pH − 0.0027 mass*mass − 0.11 pH*mass
	*R*^2^	0.8070	0.8660
	*R*^2^_adj_	0.6998	0.7915
Eu	Response reduced model	Removal (%) = 16.48 − 0.50 pH + 1.55 mass − 36.38 concentration − 0.0028 mass*mass − 0.12 pH*mass + 5.63 pH*concentration	Removal (%) = − 184.42 + 57.13 pH + 1.52 mass − 3.59 pH*pH − 0.0029 mass*mass − 0.11 pH*mass
	*R*^2^	0.8826	0.8711
	*R*^2^_adj_	0.7946	0.7994
Gd	Response reduced model	Removal (%) = 15.98 − 0.75 pH + 1.52 mass − 36.31 concentration − 0.0028 mass*mass − 0.11 pH*mass + 5.62 pH*concentration	Removal (%) = − 65.89 + 14.22 pH + 1.48 mass − 0.0027 mass*mass − 0.11 pH*mass
	*R*^2^	0.8810	0.7908
	*R*^2^_adj_	0.7918	0.7071
Tb	Response reduced model	Removal (%) = 14.32 − 1.16 pH + 1.59 mass − 36.37 concentration − 0.0033 mass*mass − 0.11 pH*mass + 5.88 pH*concentration	Removal (%) = − 67.86 + 14.72 pH + 1.55 mass − 0.0029 mass*mass − 0.12 pH*mass
	*R*^2^	0.8511	0.7820
	*R*^2^_adj_	0.7394	0.6949
Dy	Response reduced model	Removal (%) = 38.12 − 1.13 pH + 0.96 mass − 36.81 concentration − 0.11 pH*mass + 5.56 pH*concentration	Removal (%) = − 60.49 + 13.41 pH + 1.44 mass − 0.0028 mass*mass − 0.10 pH*mass
	*R*^2^	0.8233	0.8118
	*R*^2^_adj_	0.7251	0.7365

### REE removal from water and optimization of operational parameters by Response Surface Methodology

**Fig. 5:**
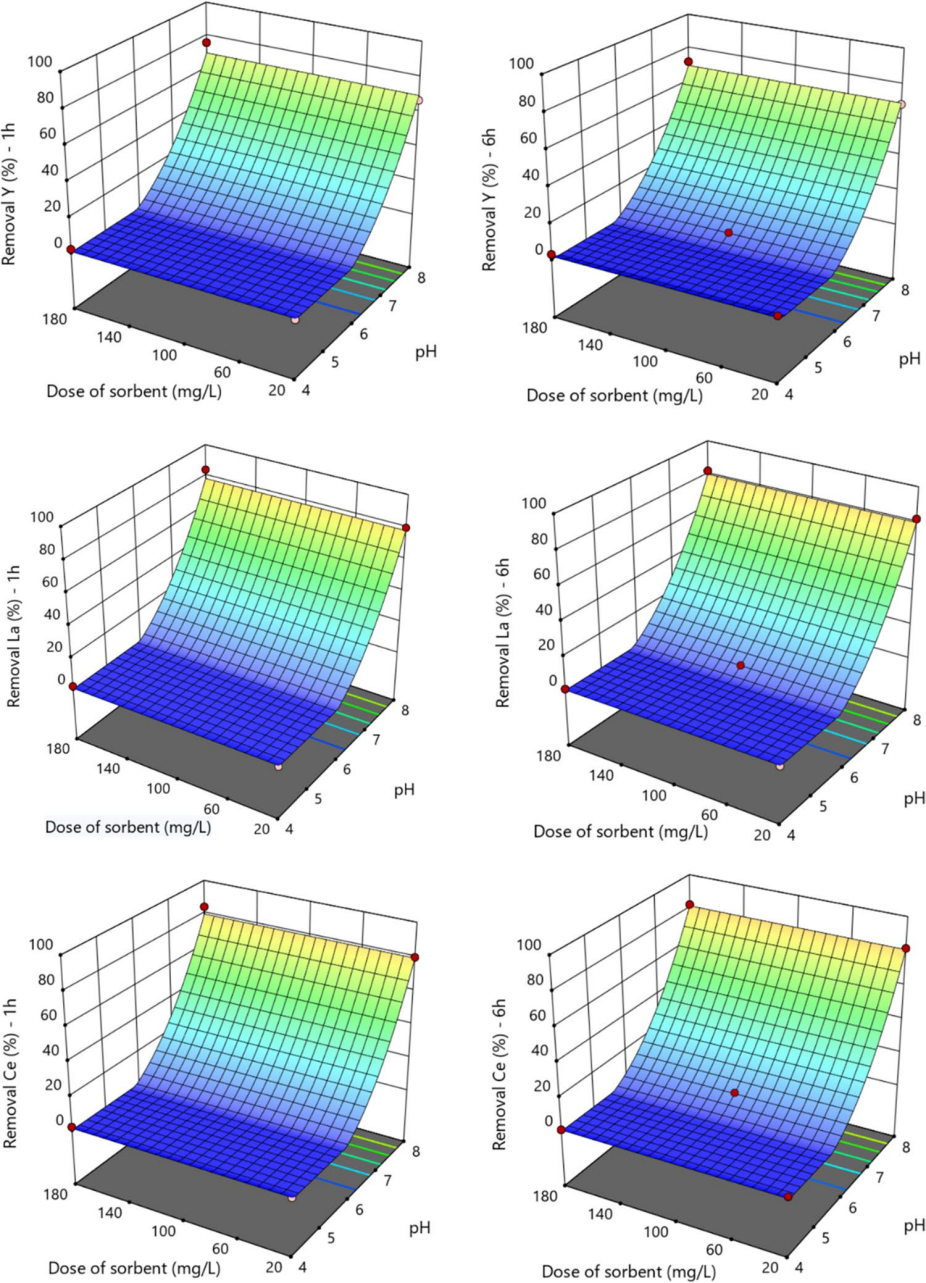
3-D response surfaces obtained with the reduced models during 1 and 6 h of exposure of Y, La, and Ce to AM-3. The figures on left present the studied response after 1 h of contact, while the figures on right present the response after 6 h, as function of the dose of sorbent and pH

Figures 5 and S3 show the 3D response surface plots for AM-3 at 1 and 6 h. The interactive effects of pH and dose of sorbent on the removal of the different REEs at the constant initial concentration of 3 μmol/L show that, independently of the dose of sorbent used, higher removal percentages are achieved at higher pH values. The interactive effects of pH and initial concentration were similar to the effects of pH and dose of sorbent and, therefore, are not shown.

**Fig. 6:**
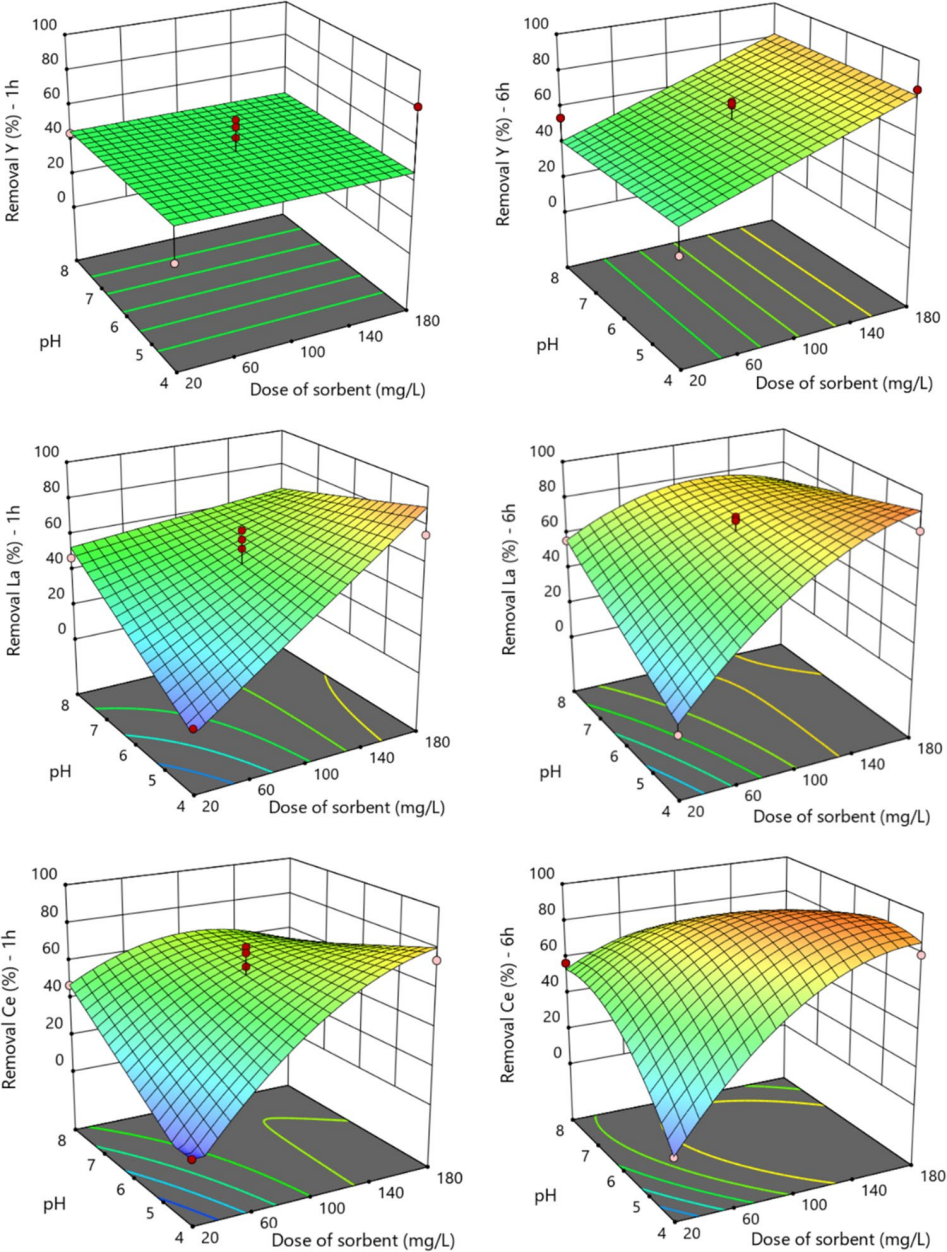
3-D response surfaces obtained with the reduced models during 1 and 6 h of exposure of Y, La, and Ce to AM-4. The figures on left present the studied response after 1 h of contact, while the figures on right present the response after 6 h, as function of the dose of sorbent and pH

Figures 6, S4, and S5 show the 3D response surface plots for AM-4, at 1 and 6 h. The interactive effects of pH and dose of sorbent on the removal at the constant initial concentration of 3 μmol/L show that the removal is more affected by the dose of sorbent than by the pH, and this effect increased with time. Concerning the interactive effects of pH and initial concentration (Figure S5), the sharp of curves demonstrate that the effect of initial concentration decreases with time.

One of the main goals of this study was to optimize the operational parameters to maximize the removal of REEs from water. The removals obtained in the experiments generated by Box-Behnken design revealed that with AM-3 there is no significant difference in the removal percentages after 1 and 6 h; likewise, the effects of independent variables do not change with time. Thus, since the reduction of sorption time is always preferable, the optimized removal time was 1 h. In contrast, the better performance of AM-4 at 6 h justifies a longer of the sorption time.

The values of the optimized variables for the REE removal by AM-3 after 1 h and by AM-4 after 6 h are presented in Table 7. The optimized values for the removal by AM-3 are: pH 8, dose of sorbent 180 mg/L and initial concentration 5 μmol/L, for a 1 h exposure, with expected removal varying between 70 and 80%. For AM-4, the optimal conditions differ only in the pH and time of exposure: pH 4.6, dose of sorbent 180 mg/L and initial concentration 5 μmol/L, for a 6 h exposure, with expected removal varying between 82 and 89%.

**Table 7:** Optimum conditions determined for REE removal with AM-3 (at 1 h) and AM-4 (at 6 h) and their removal (%) in different matrices (in natural mineral water and water with salinity 10 and with salinity 30)

Sorbent	Time (h)	REEs	Optimum conditions			% removal DoE	% removal experimental		
			pH	Dose of sorbent (mg/L)	Initial concentration (μmol/L)		Salinity 0	Salinity 10	Salinity 30
AM-3	**1**	Y	8	180	5	70	84	75	71
		La				78	93	91	89
		Ce				80	93	93	90
		Pr				78	93	92	90
		Nd				78	93	92	89
		Eu				78	93	92	89
		Gd				77	92	90	88
		Tb				78	91	89	88
		Dy				77	90	86	84
AM-4	**6**	Y	4.6	180	5	82	92	24	16
		La				89	93	14	11
		Ce				88	93	27	16
		Pr				89	93	30	15
		Nd				88	93	29	15
		Eu				88	93	41	23
		Gd				87	93	33	18
		Tb				87	93	41	23
		Dy				87	93	43	24

### Validation of the optimum conditions and the effect of salinity on REE removal

The removal percentage predictions for REEs, as determined by the DOE, were experimentally validated. To achieve this, a test was conducted under the optimal conditions obtained through the DoE (Table 7) for AM-3 at 1 h and for AM-4 at 6 h. The removal percentage results for the different REEs, as determined by DoE and obtained in the experimental test, are presented in Table 7.

The experimentally obtained removal percentages using the AM-3 sorbent for 1 h (84–93%) were higher than those predicted by the DoE (70–80%). For AM-4, the removal percentages predicted by the DoE (82–89%) were still lower than those experimentally achieved after 6 h (92–93%), but closer than in the case of AM-3. The model for AM-3 underestimated the removal obtained, while the model for AM-4 predicted the removal with an error of less than 7% for all elements, except for Y (11%).

The disparities observed between Y and the other REEs, in terms of removal percentage or the impact of the studied variables on its removal, could be attributed to the fact that Y is a transition metal and not part of the lanthanide group, which may imply distinct behaviors. According to Jacinto et al. (2018), the smaller ionic size of Y compared to other REEs appears to hinder Y’s approach to surface binding sites, due to the larger ionic size of the remaining REEs in solution.

The viability of the sorption process in more complex matrices is a crucial parameter for industry, as industrial effluents are highly complex. The removal efficiencies of AM-3 and AM-4 were assessed in real aqueous solutions with salinity 10 (common salinity in coastal and transitional systems) and salinity 30 (average salinity of seawater). The removal percentage results for the different REEs are presented in Table 7.

In general, REE removal percentages using AM-3 were high in water with salinity 10 (75–93%) and salinity 30 (71–90%). For AM-4, the removal percentages in water with salinity 10 ranged between 14 and 43%, decreasing further in water with salinity 30 (11–24%).

It is evident that titanosilicate AM-3 possesses strong potential for REE removal in intermediate salinity water (salinity 10) and in water with seawater salinity (salinity 30). Conversely, the REE removal efficiency of AM-4 significantly declined with increasing salinity, suggesting competition for binding sites with other ions present in the solution, such as Na^+^, Ca^2+^, and Mg^2+^ (which are the dominant cations in this matrix). These differences between the two titanosilicates are likely related to their structure, as AM-3 is a nanoporous titanosilicate and AM-4 is a layered titanosilicate. According to Oleksiienko et al. (2017), AM-4 exhibits low affinity for alkali cations (Na^+^) but high affinity for alkaline earth metals (Ca^2+^ and Mg^2+^) in neutral and alkaline media.

This study demonstrates that, in terms of applicability in aquatic systems or natural waters, the use of AM-3 may be more advantageous compared to AM-4.

## Conclusion

The application of Design of Experiments enabled the identification of the most critical variables in these processes with minimal experimental efforts and optimized the removal of nine different REEs using AM-3 and AM-4. The structural differences between AM-3 (nanoporous titanosilicate) and AM-4 (layered titanosilicate) resulted in variations in the influence of variables on sorption. The pH had the most significant impact on AM-3’s removal efficiency, with higher performance at increased pH levels due to competition between H^+^ and REE^3+^ ions for the exchange of the extra-framework Na^+^ cations. In contrast, AM-4’s removal efficiency was primarily affected by the dose of sorbent, with higher doses resulting in greater removals.

Response Surface Methodology proved to be a valuable tool for gaining insights into the behavior of REE removal processes and the optimal responses expected within various operational conditions. The performance of the two titanosilicates under optimal conditions demonstrated that both materials have strong potential for this removal process. However, when considering process viability in more complex matrices, the two sorbents exhibit different behaviors. The influence of salinity on AM-3’s removal efficiency did not negatively impact its performance, unlike AM-4, which exhibited poor performance in the presence of salinity due to competition between REEs and other cations in the solution. Consequently, it is concluded that the AM-3 sorbent material is the superior choice for potential applications in real aqueous systems, where the presence of competitive ions is more common.

### Supplementary Information

Below is the link to the electronic supplementary material.

Supplementary file1 (DOCX 10013 KB)
