# Supplementary material for: Enhanced removal of rare earth elements from aqueous media: exploring the potential of AM-3 and AM-4 titanosilicates
Source: Environ Sci Pollut Res Int. 2024 Apr 2;31(20):28856–69. doi: 10.1007/s11356-024-33063-w (PMC11058749; doi:10.1007/s11356-024-33063-w)
Supplement: Supplementary file 1 — Supplementary file1 (DOCX 10013 KB) [file 11356_2024_33063_MOESM1_ESM.docx]

**Supplementary information**

**Waters composition**

Natural mineral water data:

pH – 5,2 (±0,4)

Total mineralization - 32 (±2) mg/L

Silica – 9,0 (±0,4) mg/L

Chlorides – 9,6 (±0,4) mg/L

Bicarbonates – 2,9 (±0,5) mg/L

Sulfates – 1,4 (±0,2) mg/L

Nitrates – 1,9 (±0,2) mg/L

Sodium – 5,6 (±0,4) mg/L

Magnesium – 1,0 (±0,2) mg/L

Calcium – 0,6 (±0,2) mg/L

Real seawater data:

The seawater was collected in Costa Nova, Ílhavo, at the shore of the Portuguese coast, at the Atlantic Ocean (40º36′55″N, 8º45′22″W).

pH – 8,1

Dissolved organic content < 1 mg/L

Suspended particle matter - 20,5 mg/L

Sodium > 1000 mg/L

Potassium > 600 mg/L

Calcium > 500 mg/L

Magnesium > 250 mg/L

**Figures**


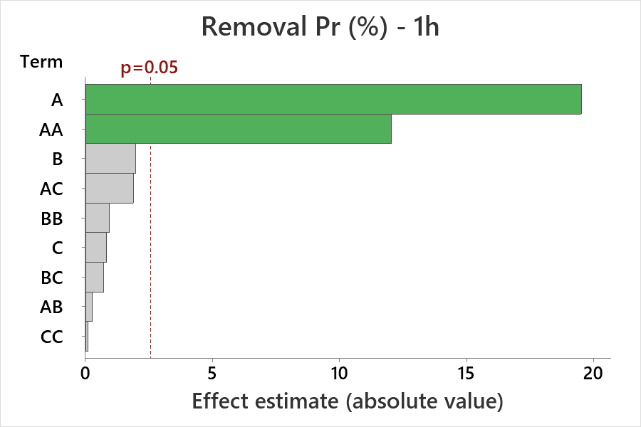

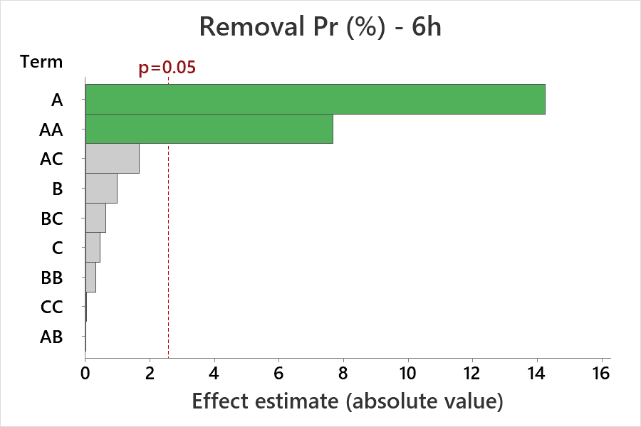

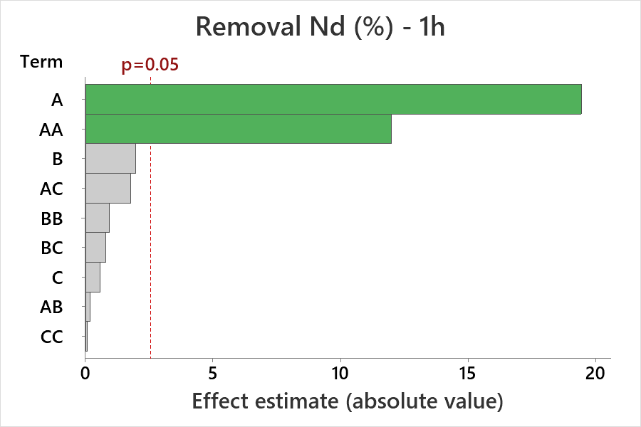

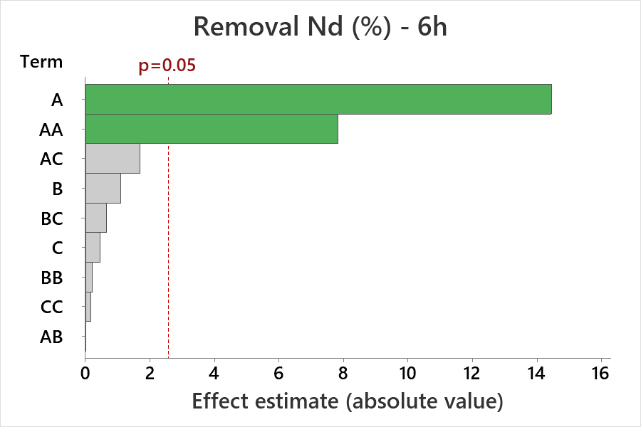

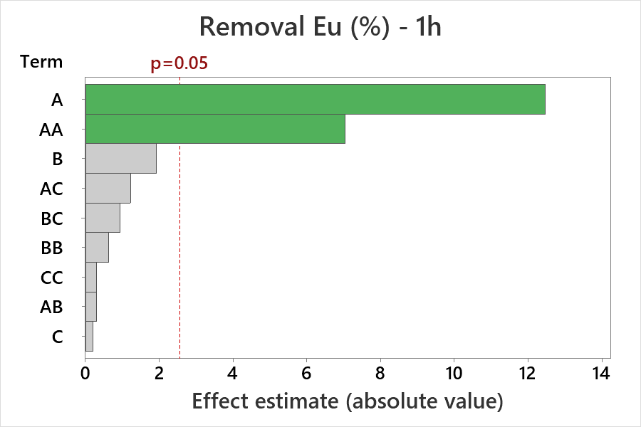

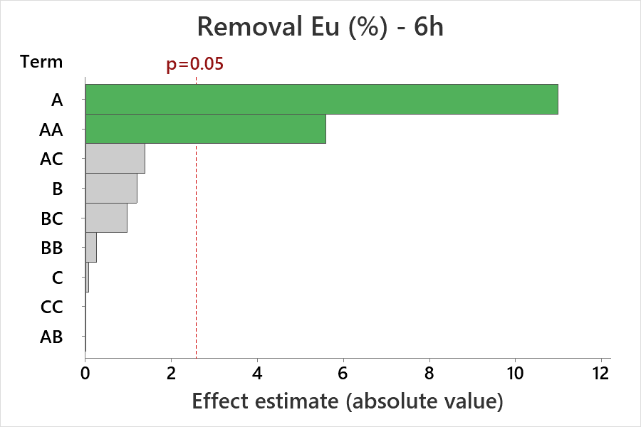

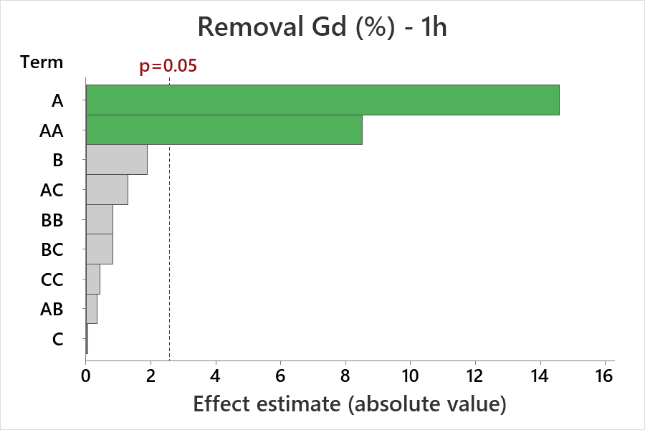

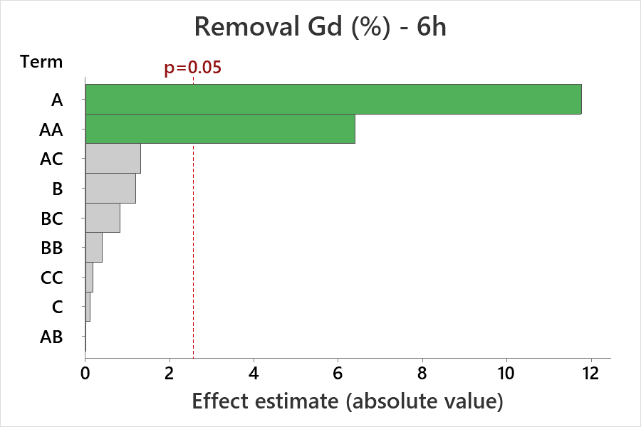

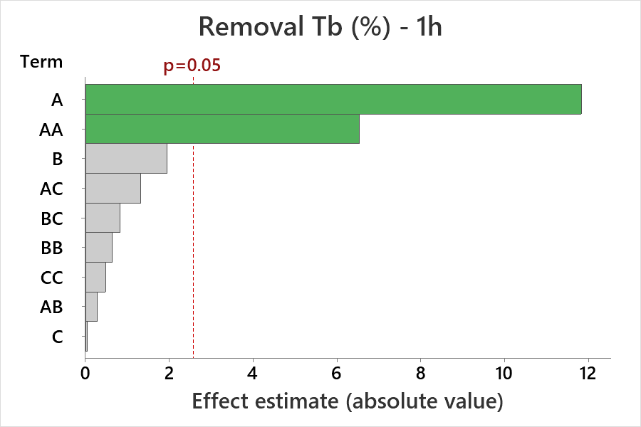

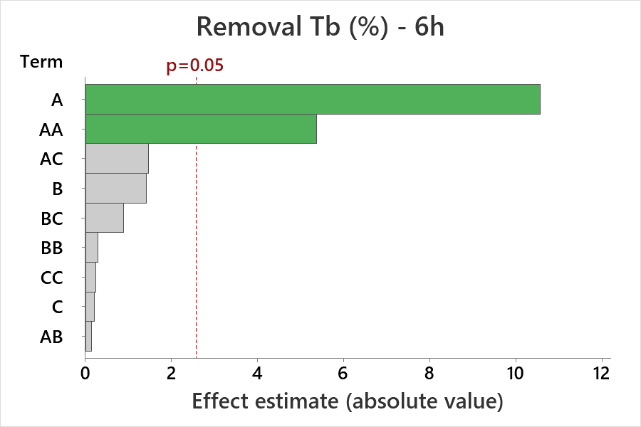

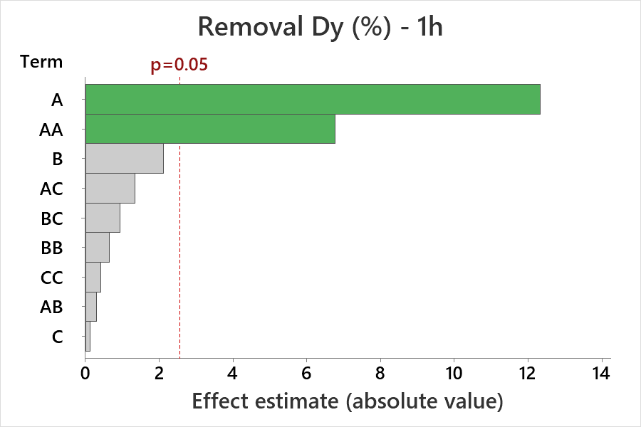

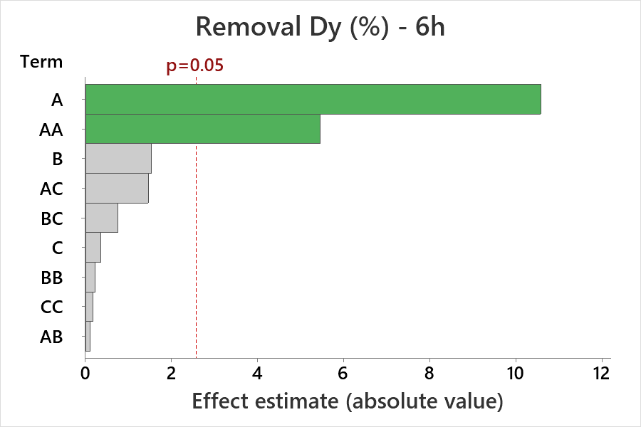


Figure S1 - Pareto chart displaying the effects of variables on the studied response (removal percentage of Pr, Nd, Eu, Gd, Tb and Dy) at 1 and 6 hours for AM-3. In the figure: A represents the solution pH, B denotes the sorbent dosage (mg/L), and C signifies the initial concentration of REEs (µmol/L). Variables with values below the dashed line are not significant.


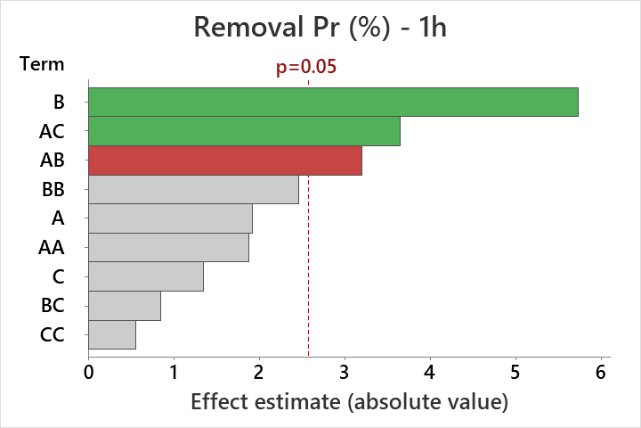

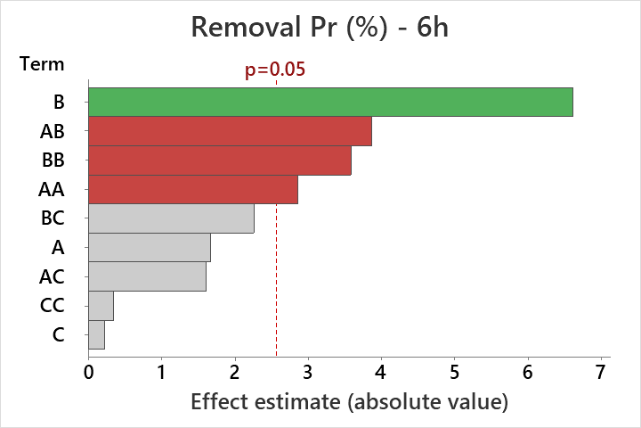

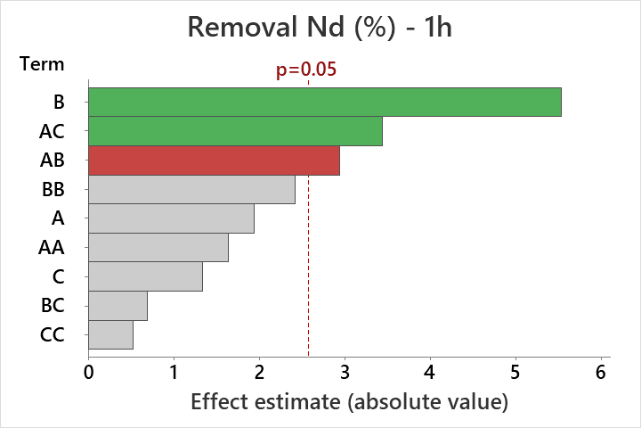

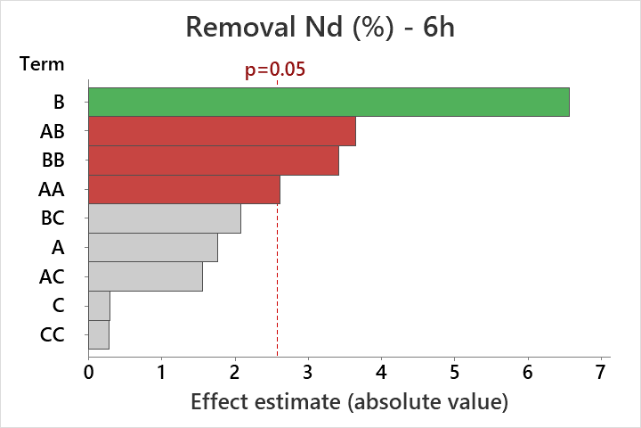

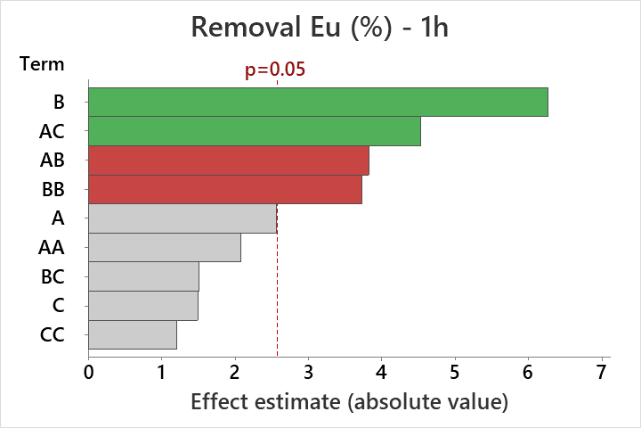

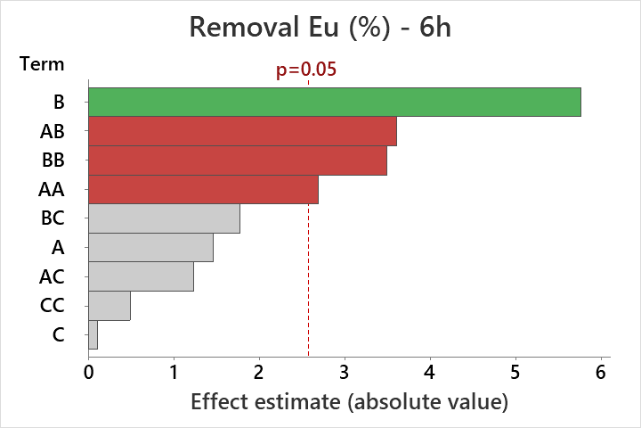

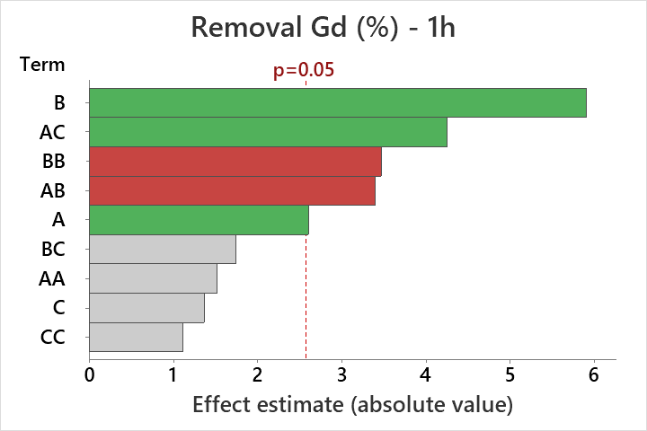

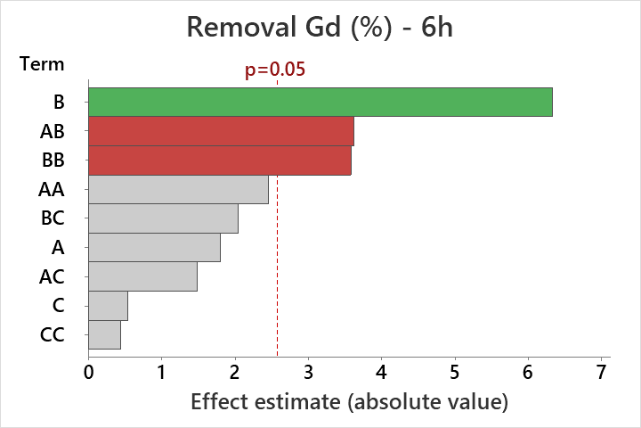

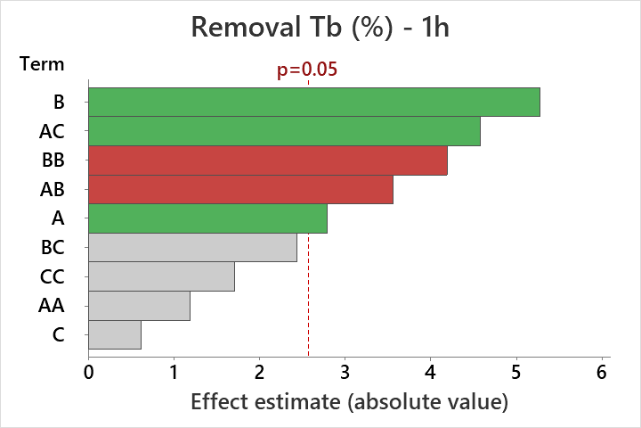

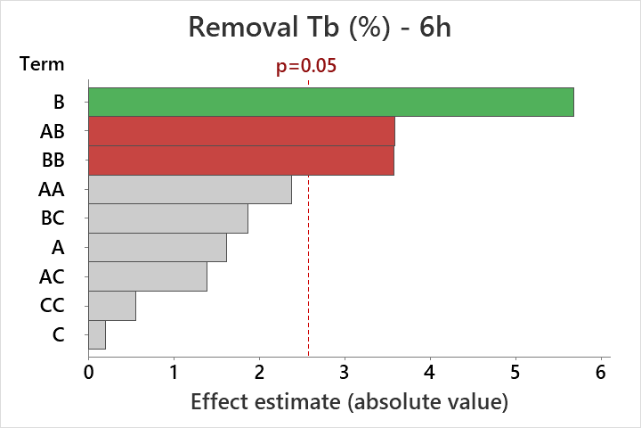

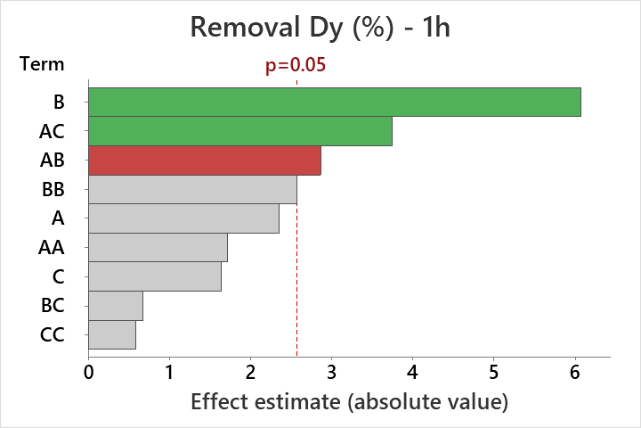

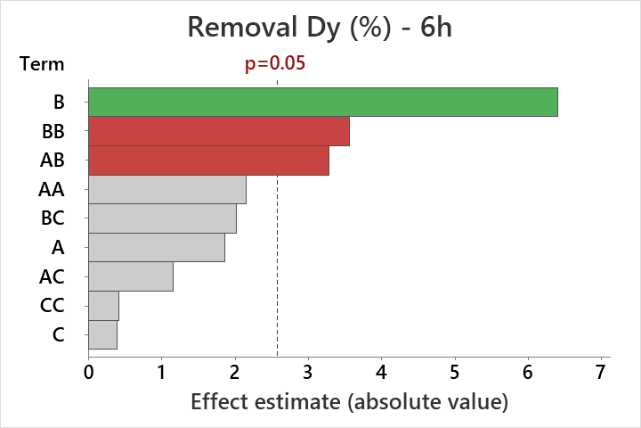


Figure S2 - Pareto chart displaying the effects of variables on the studied response (removal percentage of Pr, Nd, Eu, Gd, Tb and Dy) at 1 and 6 hours for AM-4. In the figure: A represents the solution pH, B denotes the sorbent dosage (mg/L), and C signifies the initial concentration of REEs (µmol/L). Variables with values below the dashed line are not significant.


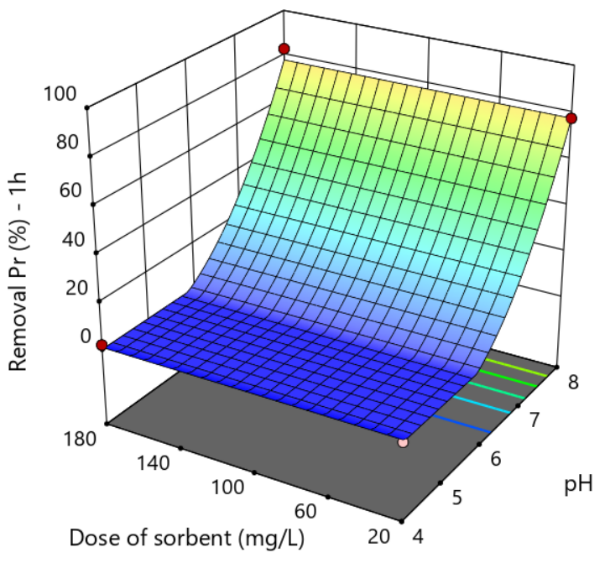

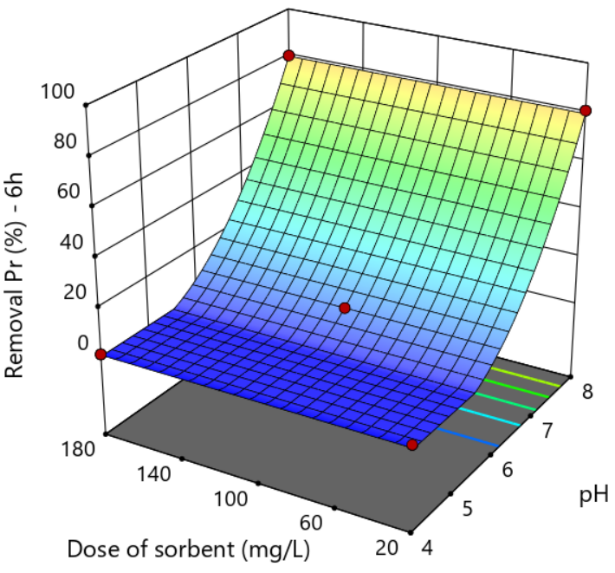


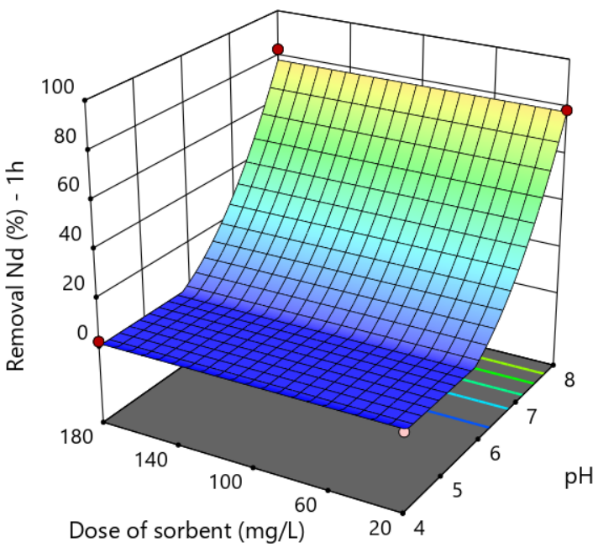

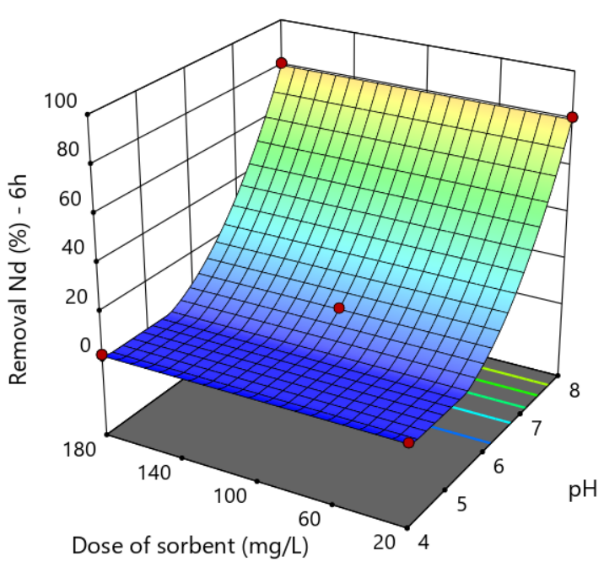


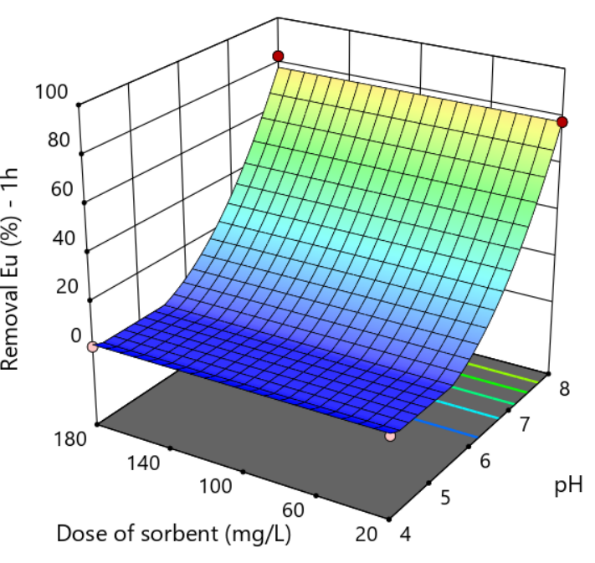

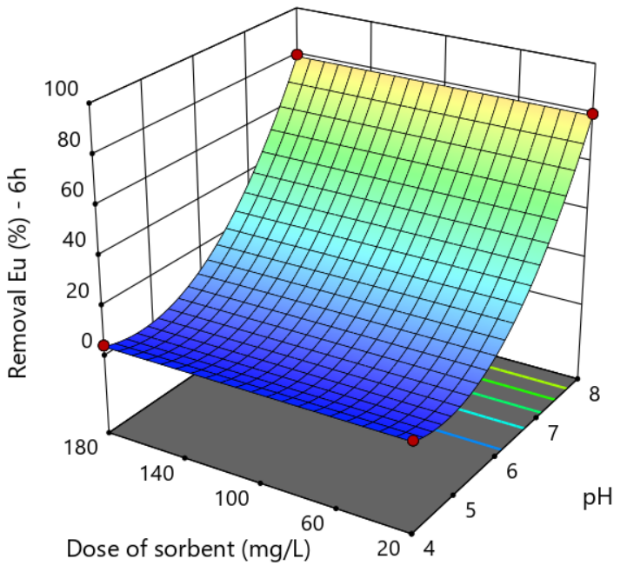


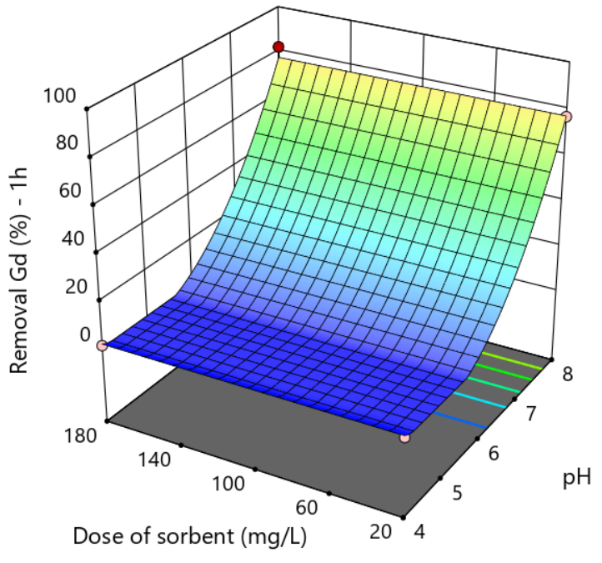

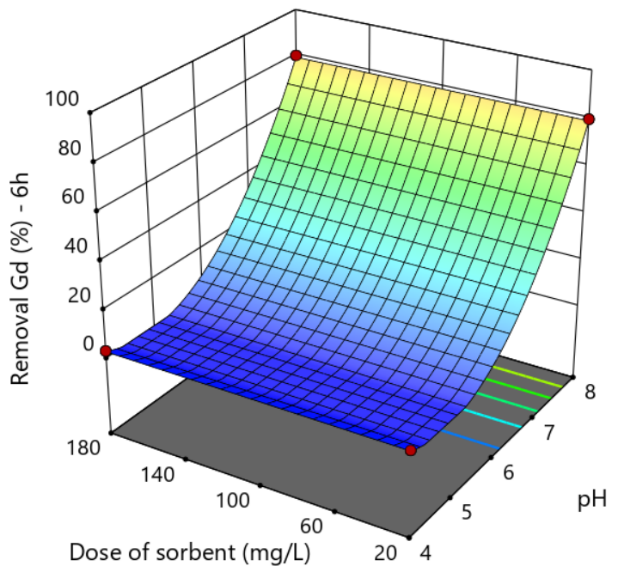


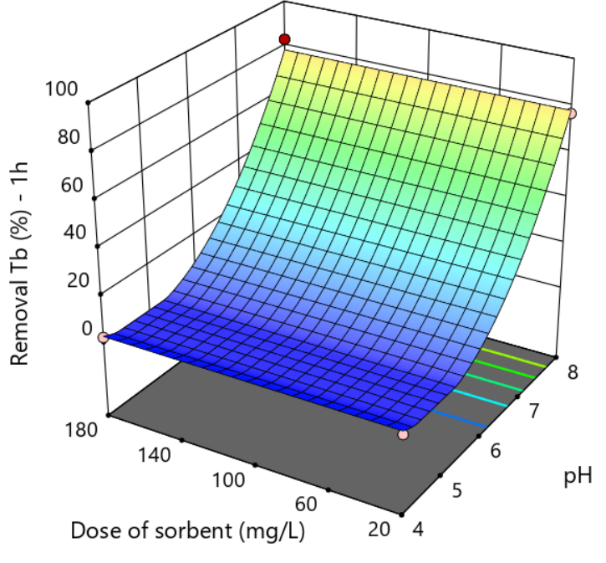

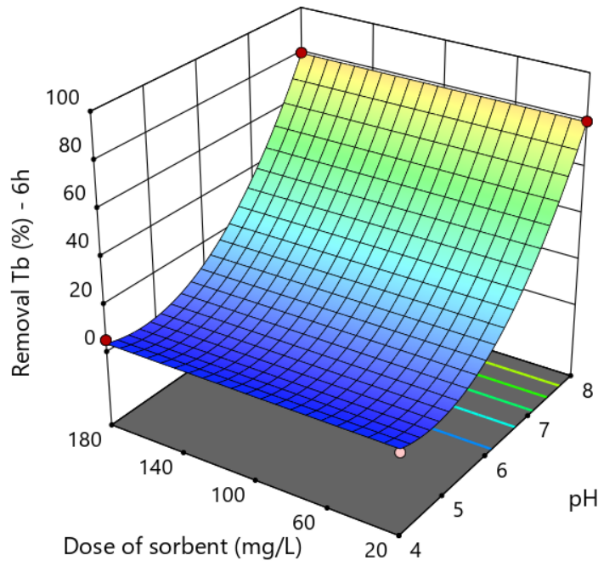


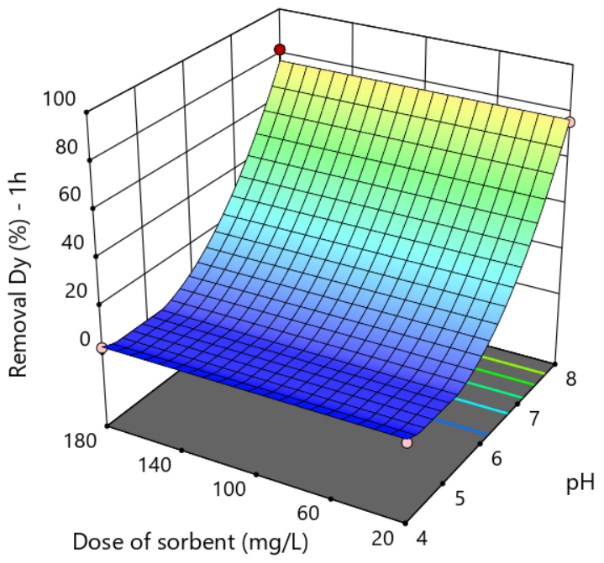

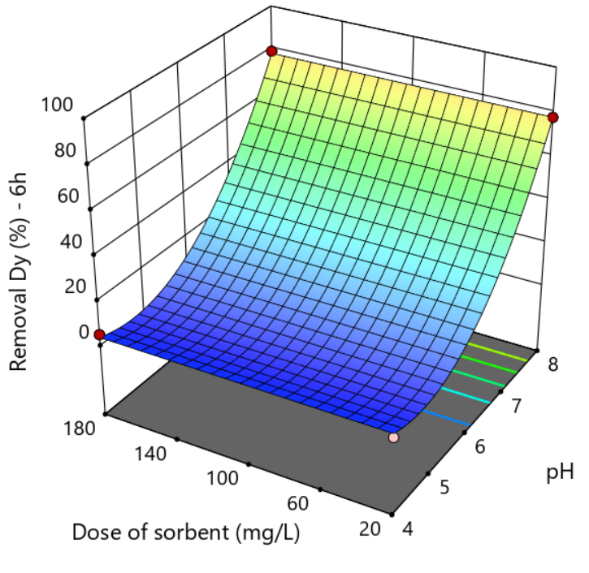


Figure S3 - 3-D Response Surfaces obtained with the reduced models during 1 and 6 hours of exposure of Pr, Nd, Eu, Gd, Tb and Dy to AM-3. The figures on left present the studied response after 1 hour of contact, while the figures on right present the response after 6 hours, as function of the dose of sorbent and pH.


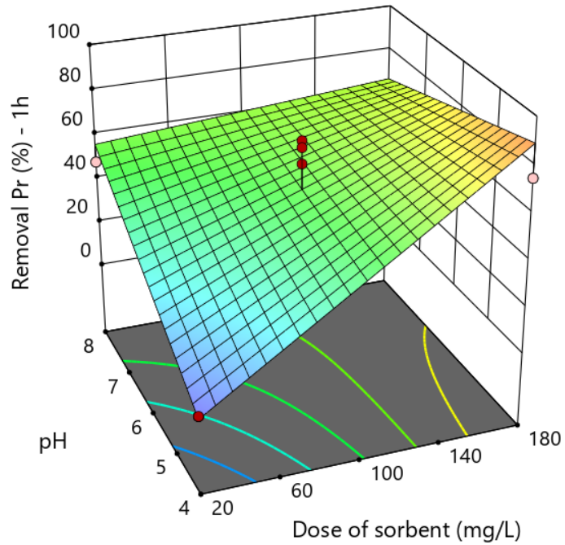

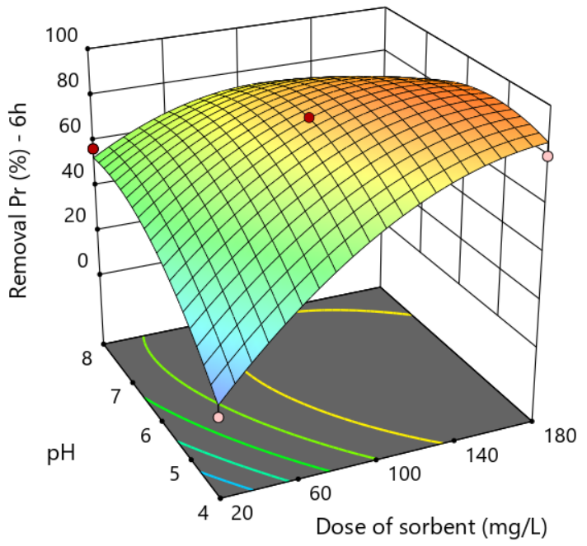


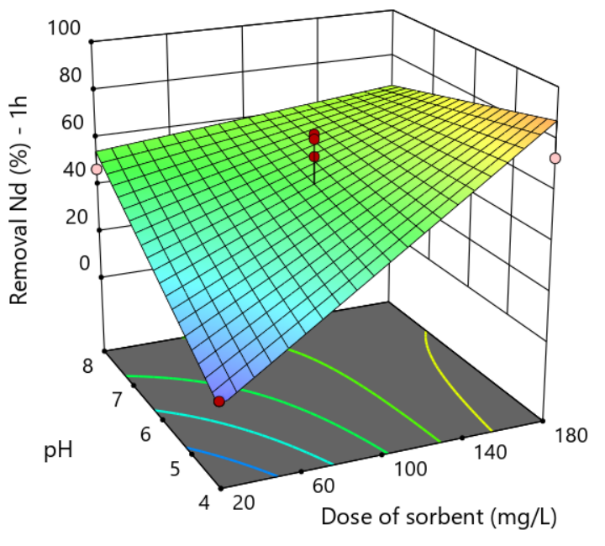

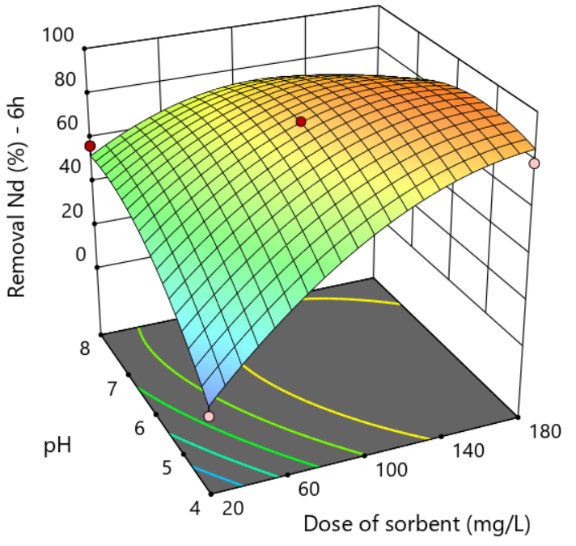


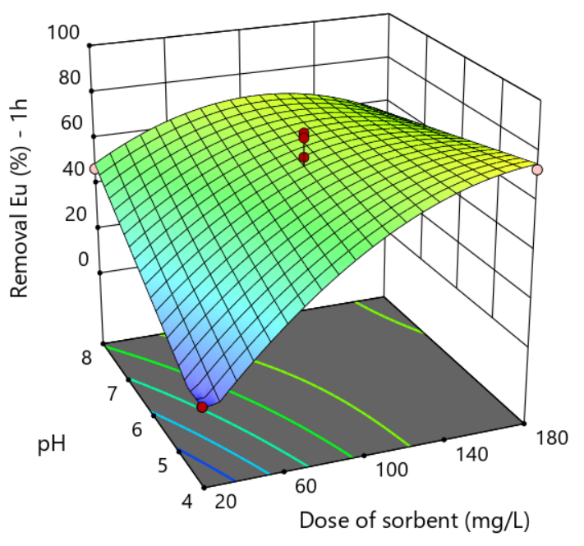

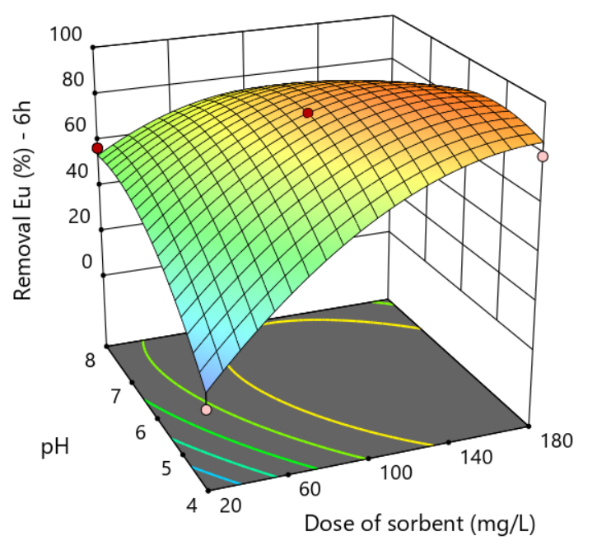


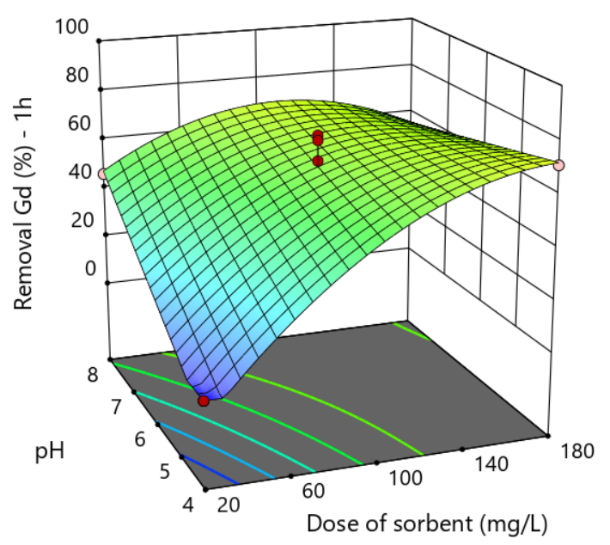

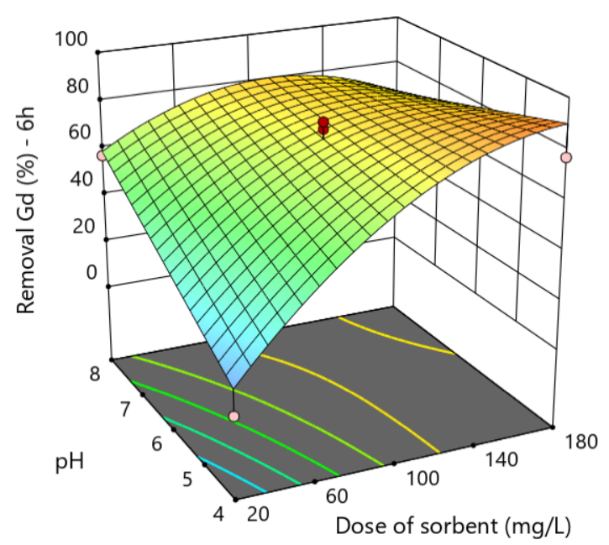


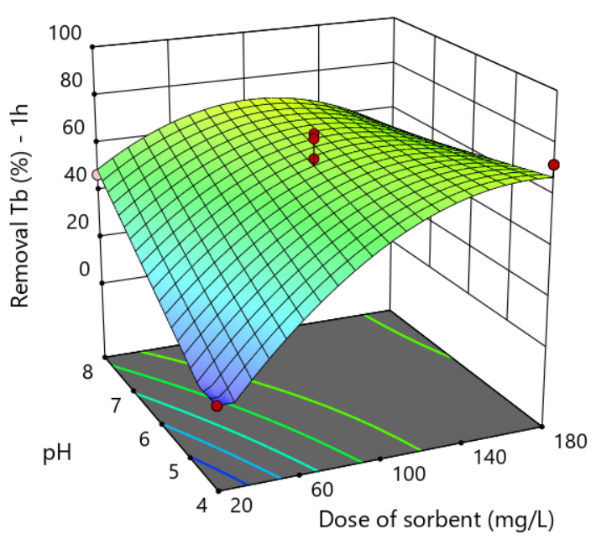

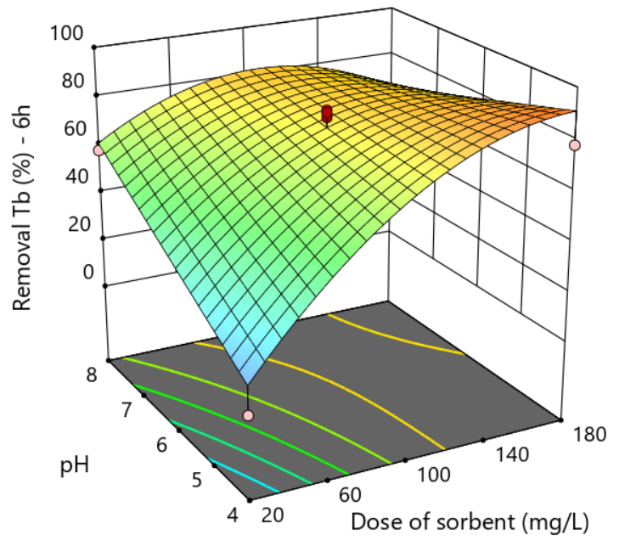


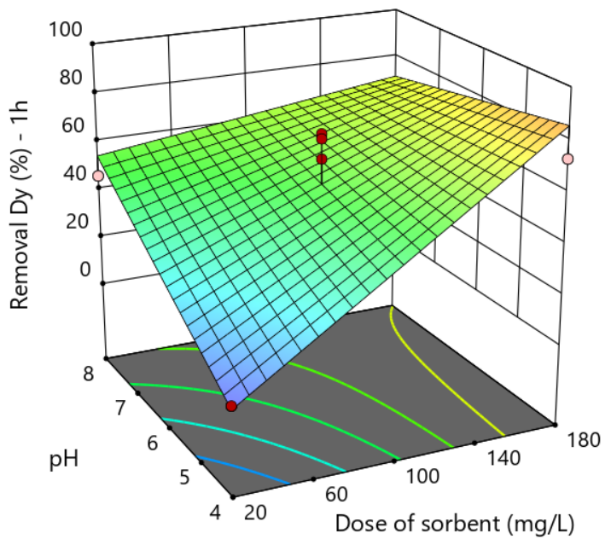

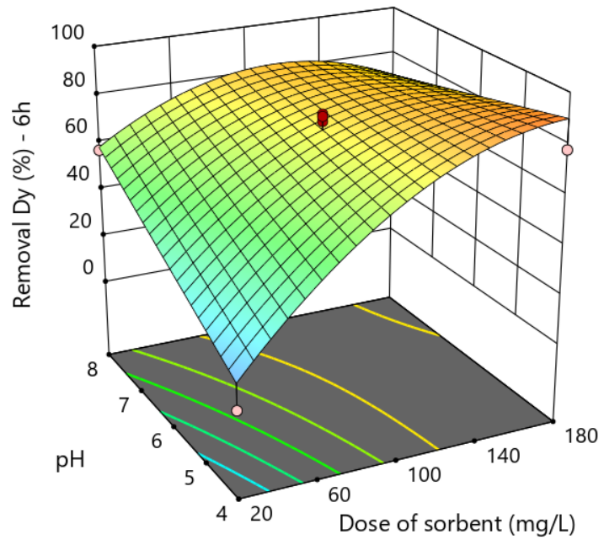


Figure S4 - 3-D Response Surfaces obtained with the reduced models during 1 and 6 hours of exposure of Pr, Nd, Eu, Gd, Tb and Dy to AM-4. The figures on left present the studied response after 1 hour of contact, while the figures on right present the response after 6 hours, as function of the dose of sorbent and pH.


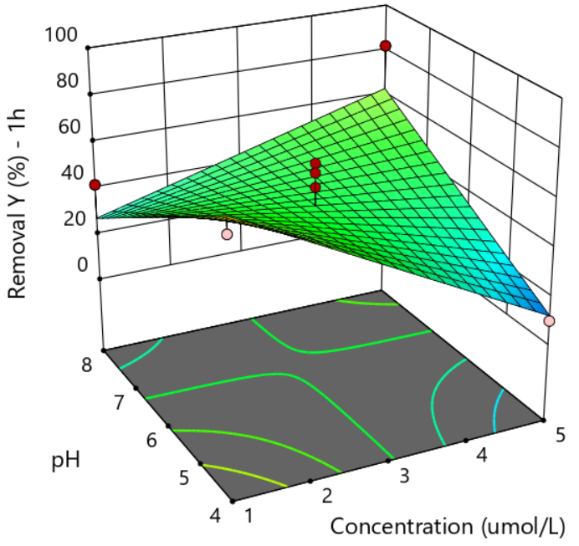

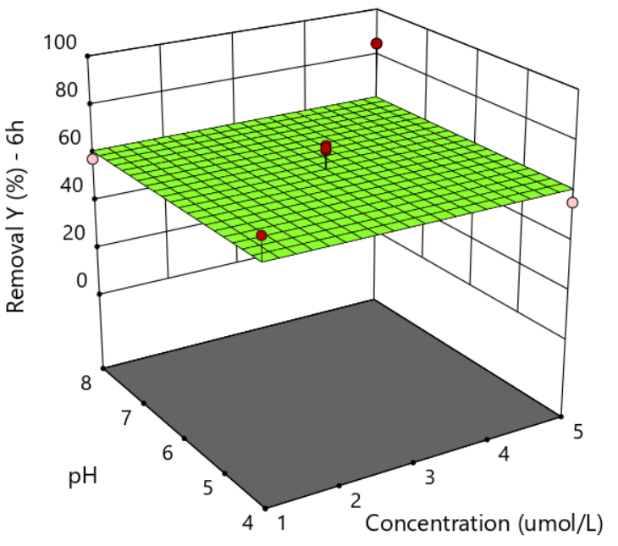


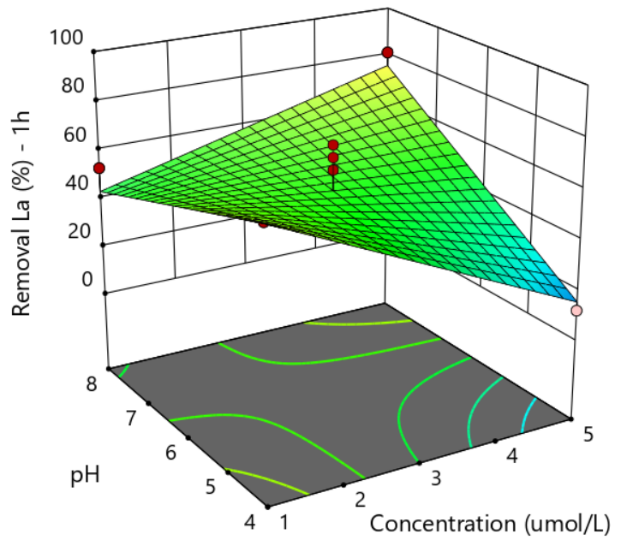

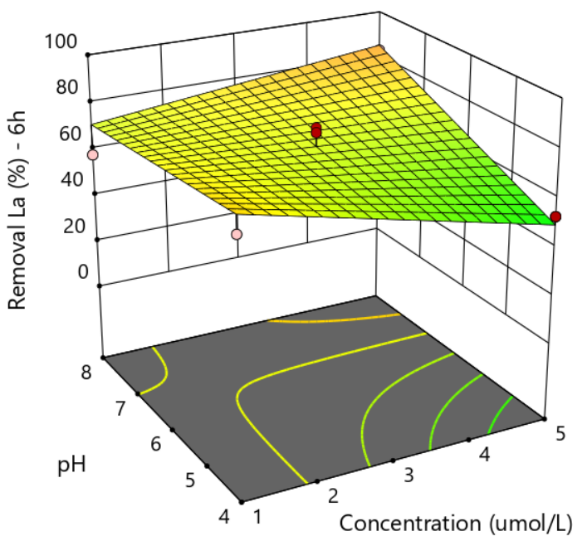


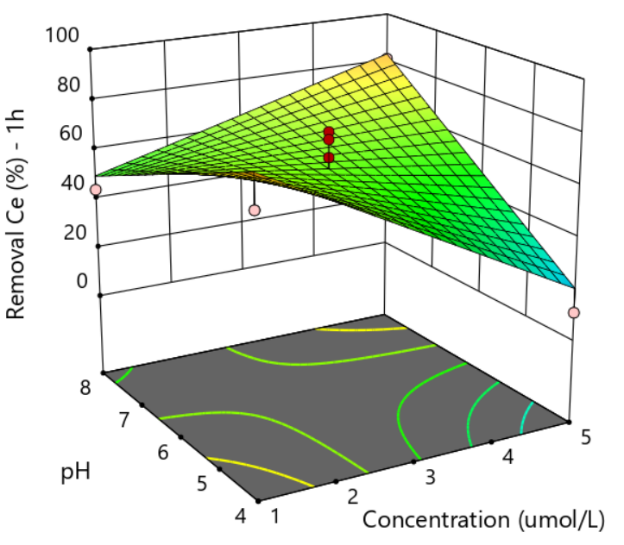

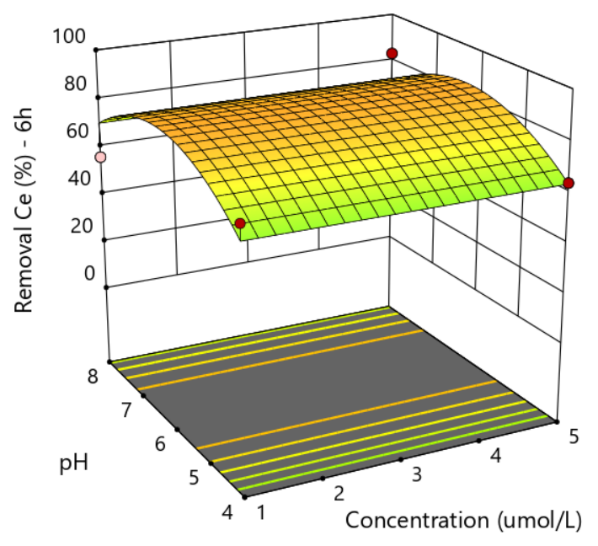


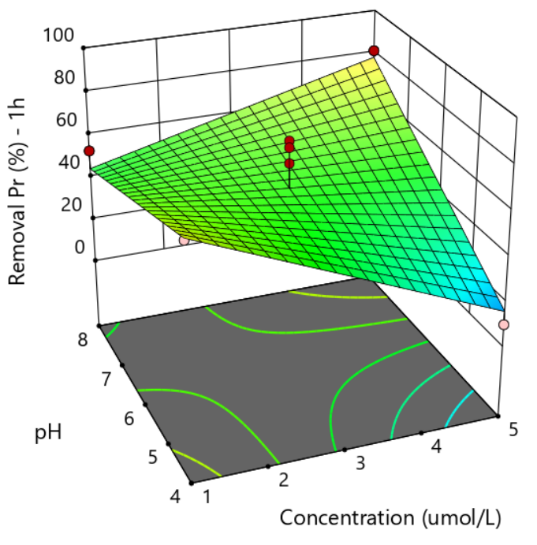

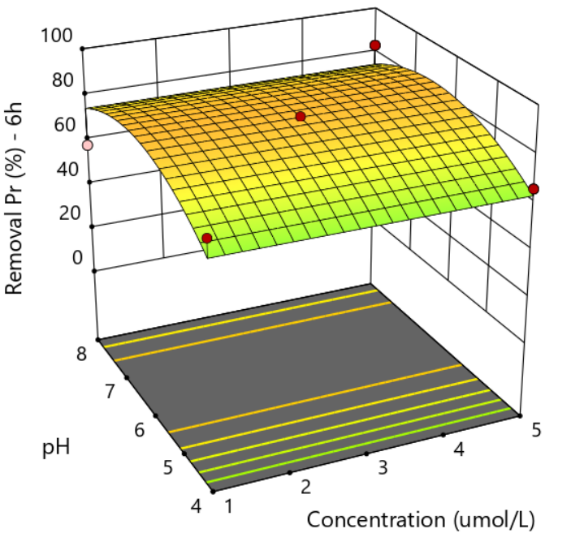


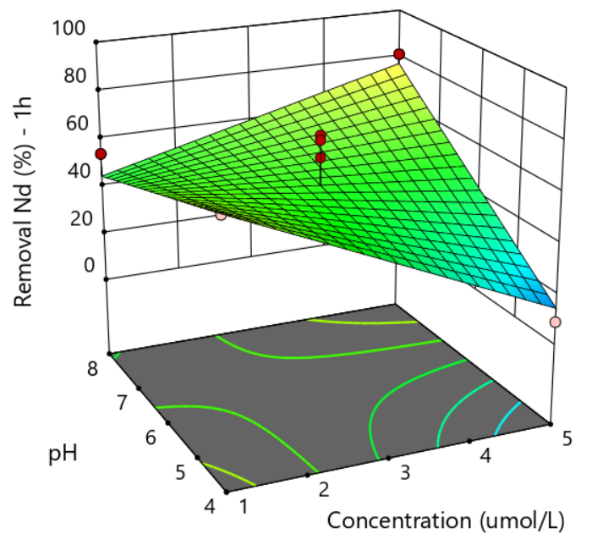

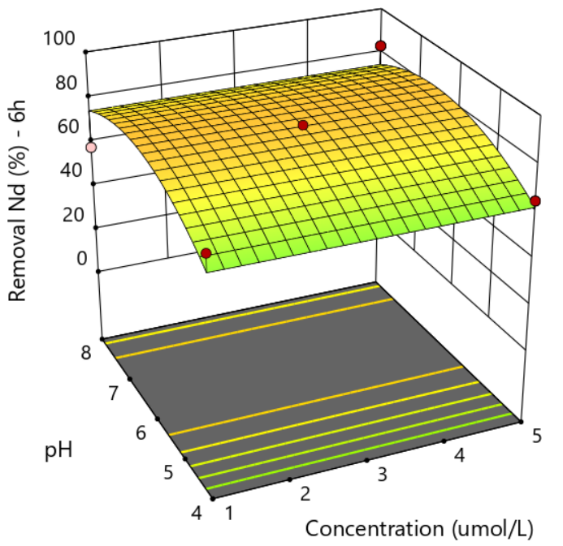


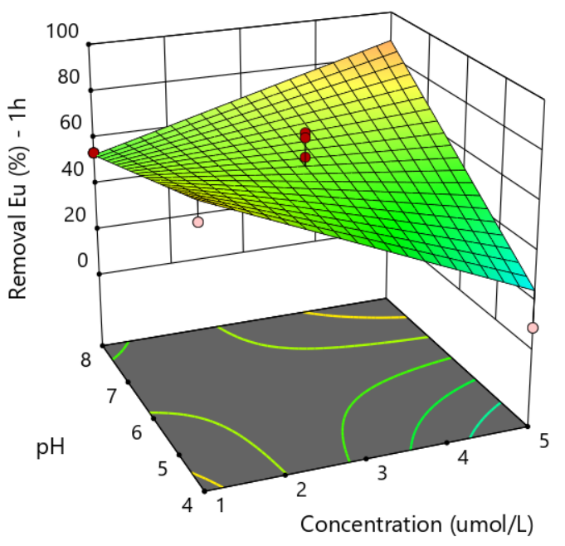

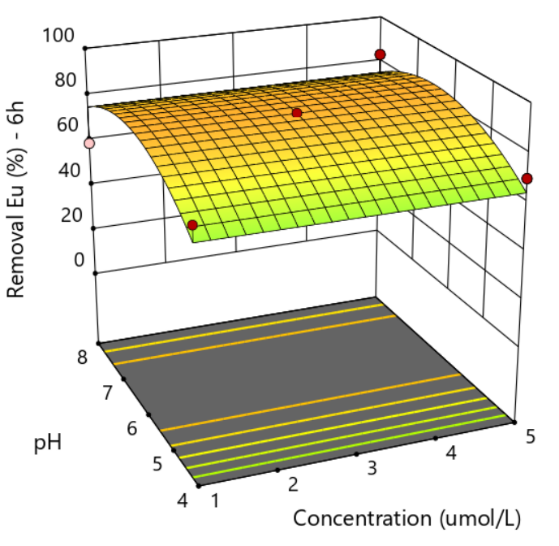


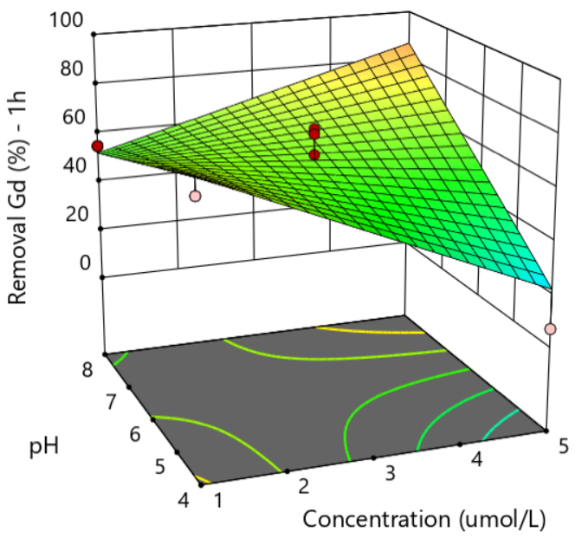

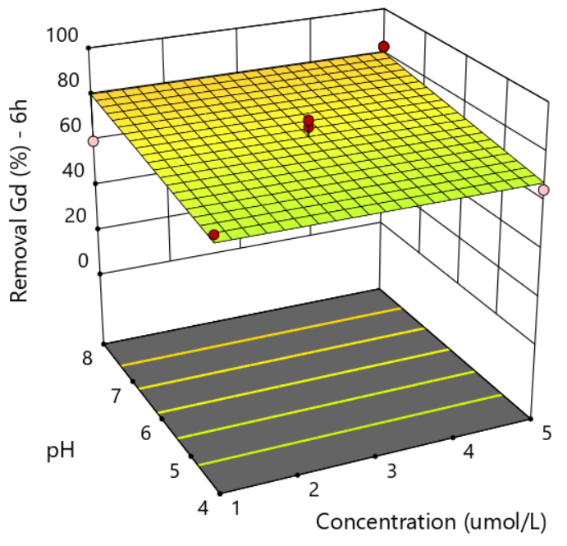


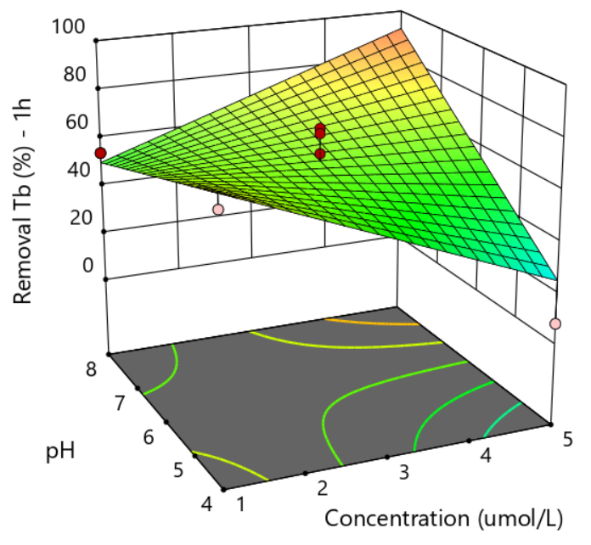

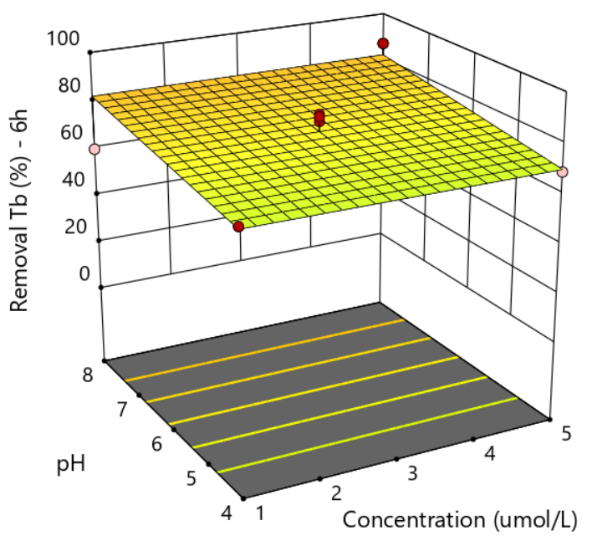


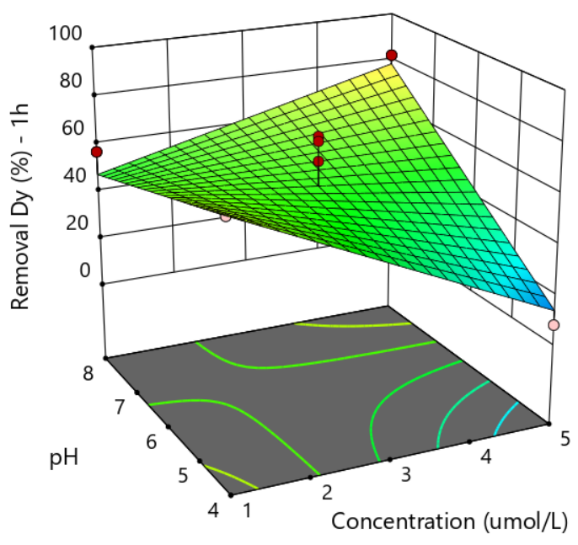

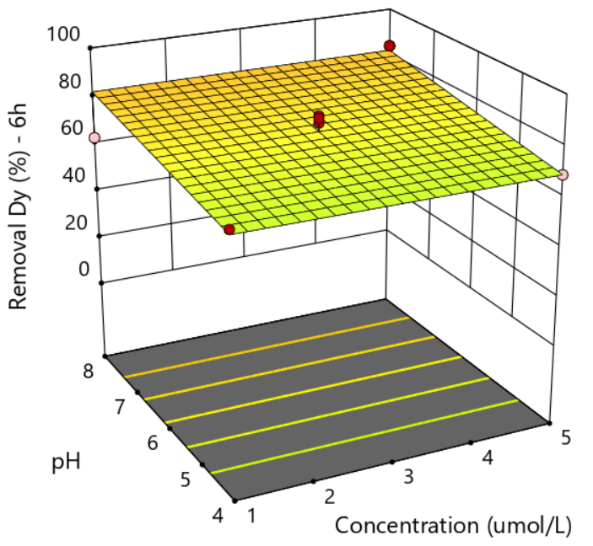


Figure S5 - 3-D Response Surfaces obtained with the reduced models during 1 and 6 hours of exposure of Y, La, Ce, Pr, Nd, Eu, Gd, Tb and Dy to AM-4. The figures on left present the studied response after 1 hour of contact, while the figures on right present the response after 6 hours, as function of the initial concentration of REEs and pH.

**Tables**

Table S1 - p-value for each factor considered by the mathematical model for removal % of Y, La Ce, Pr, Nd, Eu, Gd, Tb and Dy using the titanosilicate AM-3.

|  |  | **Y** | | **La** | | **Ce** | | **Pr** | | **Nd** | |
| --- | --- | --- | --- | --- | --- | --- | --- | --- | --- | --- | --- |
|  |  | **1h** | **6h** | **1h** | **6h** | **1h** | **6h** | **1h** | **6h** | **1h** | **6h** |
| p-value | A | < 0.0001 | < 0.0001 | < 0.0001 | < 0.0001 | < 0.0001 | < 0.0001 | < 0.0001 | < 0.0001 | < 0.0001 | < 0.0001 |
|  | B | 0.0926 | 0.2596 | 0.1829 | 0.3634 | 0.1231 | 0.3057 | 0.1068 | 0.3669 | 0.1078 | 0.3201 |
|  | C | 0.1842 | 0.2083 | 0.2209 | 0.2763 | 0.5231 | 0.6867 | 0.4477 | 0.6711 | 0.5946 | 0.6650 |
|  | AB | 0.5368 | 0.8161 | 0.8433 | 0.9404 | 0.7827 | 0.9490 | 0.7989 | 1.0000 | 0.8653 | 1.0000 |
|  | AC | 0.0642 | 0.0870 | 0.0546 | 0.0875 | 0.1411 | 0.1410 | 0.1187 | 0.1584 | 0.1343 | 0.1517 |
|  | BC | 0.7204 | 0.8161 | 0.7675 | 0.8231 | 0.4727 | 0.5717 | 0.5056 | 0.5519 | 0.4583 | 0.5442 |
|  | A² | < 0.0001 | 0.0002 | < 0.0001 | 0.0001 | < 0.0001 | 0.0004 | < 0.0001 | 0.0006 | < 0.0001 | 0.0005 |
|  | B² | 0.3764 | 0.6575 | 0.2557 | 0.6782 | 0.3726 | 0.8065 | 0.3872 | 0.7572 | 0.3955 | 0.8433 |
|  | C² | 0.8180 | 0.9405 | 0.7643 | 0.8765 | 0.9646 | 0.9510 | 0.9347 | 0.9690 | 0.9458 | 0.8742 |

|  |  | **Eu** | | **Gd** | | **Tb** | | **Dy** | |
| --- | --- | --- | --- | --- | --- | --- | --- | --- | --- |
|  |  | **1h** | **6h** | **1h** | **6h** | **1h** | **6h** | **1h** | **6h** |
| p-value | A | < 0.0001 | 0.0001 | < 0.0001 | < 0.0001 | < 0.0001 | 0.0001 | < 0.0001 | 0.0001 |
|  | B | 0.1118 | 0.2849 | 0.1183 | 0.2868 | 0.1085 | 0.2146 | 0.0886 | 0.1863 |
|  | C | 0.8456 | 0.9450 | 0.9633 | 0.9127 | 0.9704 | 0.8435 | 0.9060 | 0.7363 |
|  | AB | 0.7834 | 1.0000 | 0.7466 | 1.0000 | 0.7936 | 0.8889 | 0.7816 | 0.9237 |
|  | AC | 0.2775 | 0.2246 | 0.2510 | 0.2487 | 0.2426 | 0.2014 | 0.2361 | 0.2040 |
|  | BC | 0.3959 | 0.3744 | 0.4497 | 0.4518 | 0.4454 | 0.4181 | 0.3920 | 0.4841 |
|  | A² | 0.0009 | 0.0025 | 0.0004 | 0.0014 | 0.0013 | 0.0030 | 0.0011 | 0.0028 |
|  | B² | 0.5553 | 0.8031 | 0.4437 | 0.7113 | 0.5525 | 0.7832 | 0.5520 | 0.8303 |
|  | C² | 0.7782 | 0.9875 | 0.6800 | 0.8621 | 0.6533 | 0.8175 | 0.6972 | 0.8662 |

Table S2 - p-value for each factor considered by the mathematical model for removal % of Y, La Ce, Pr, Nd, Eu, Gd, Tb and Dy using the titanosilicate AM-4.

|  |  | **Y** | | **La** | | **Ce** | | **Pr** | | **Nd** | |
| --- | --- | --- | --- | --- | --- | --- | --- | --- | --- | --- | --- |
|  |  | **1h** | **6h** | **1h** | **6h** | **1h** | **6h** | **1h** | **6h** | **1h** | **6h** |
| p-value | A | 0.9671 | 1.0000 | 0.1656 | 0.0683 | 0.1295 | 0.3790 | 0.1126 | 0.1549 | 0.1096 | 0.1368 |
|  | B | 0.0627 | 0.0101 | 0.0027 | 0.0004 | 0.0015 | 0.0024 | 0.0023 | 0.0012 | 0.0027 | 0.0012 |
|  | C | 0.2364 | 0.6301 | 0.2409 | 0.2409 | 0.0918 | 0.7000 | 0.2354 | 0.8285 | 0.2376 | 0.7797 |
|  | AB | 0.0576 | 0.0555 | 0.0325 | 0.0081 | 0.0115 | 0.0046 | 0.0239 | 0.0117 | 0.0322 | 0.0147 |
|  | AC | 0.0260 | 0.2068 | 0.0204 | 0.0443 | 0.0056 | 0.1439 | 0.0147 | 0.1674 | 0.0183 | 0.1783 |
|  | BC | 0.9071 | 0.1979 | 0.4265 | 0.0270 | 1.0000 | 0.0690 | 0.4331 | 0.0733 | 0.5171 | 0.0912 |
|  | A² | 0.8374 | 0.5464 | 0.2616 | 0.0509 | 0.0786 | 0.0174 | 0.1183 | 0.0354 | 0.1614 | 0.0469 |
|  | B² | 0.0917 | 0.1305 | 0.0861 | 0.0145 | 0.0237 | 0.0089 | 0.0566 | 0.0158 | 0.0599 | 0.0187 |
|  | C² | 0.3537 | 0.7655 | 0.8369 | 0.9631 | 0.3549 | 0.5820 | 0.6005 | 0.7444 | 0.6209 | 0.7877 |

|  |  | **Eu** | | **Gd** | | **Tb** | | **Dy** | |
| --- | --- | --- | --- | --- | --- | --- | --- | --- | --- |
|  |  | **1h** | **6h** | **1h** | **6h** | **1h** | **6h** | **1h** | **6h** |
| p-value | A | 0.0503 | 0.2022 | 0.0480 | 0.1305 | 0.0383 | 0.1652 | 0.0653 | 0.1219 |
|  | B | 0.0015 | 0.0022 | 0.0020 | 0.0015 | 0.0033 | 0.0023 | 0.0017 | 0.0014 |
|  | C | 0.1949 | 0.9206 | 0.2294 | 0.6109 | 0.5620 | 0.8471 | 0.1622 | 0.7102 |
|  | AB | 0.0123 | 0.0154 | 0.0193 | 0.0151 | 0.0162 | 0.0157 | 0.0352 | 0.0218 |
|  | AC | 0.0062 | 0.2716 | 0.0081 | 0.1983 | 0.0059 | 0.2238 | 0.0133 | 0.2971 |
|  | BC | 0.1911 | 0.1354 | 0.1411 | 0.0963 | 0.0588 | 0.1209 | 0.5302 | 0.0989 |
|  | A² | 0.0919 | 0.0433 | 0.1891 | 0.0575 | 0.2858 | 0.0629 | 0.1460 | 0.0837 |
|  | B² | 0.0136 | 0.0173 | 0.0178 | 0.0158 | 0.0085 | 0.0159 | 0.0500 | 0.0161 |
|  | C² | 0.2803 | 0.6445 | 0.3170 | 0.6769 | 0.1480 | 0.6000 | 0.5828 | 0.6911 |
